# Transcription/Replication Conflicts in Tumorigenesis and Their Potential Role as Novel Therapeutic Targets in Multiple Myeloma

**DOI:** 10.3390/cancers13153755

**Published:** 2021-07-27

**Authors:** Laure Dutrieux, Yea-Lih Lin, Malik Lutzmann, Raphaël Rodriguez, Michel Cogné, Philippe Pasero, Jérôme Moreaux

**Affiliations:** 1 Institute of Human Genetics, UMR 9002 Centre National de la Recherche Scientifique, University of Montpellier, 34396 Montpellier, France; laure.dutrieux@igh.cnrs.fr (L.D.); Yea-Lih.Lin@igh.cnrs.fr (Y.-L.L.); malik.lutzmann@igh.cnrs.fr (M.L.); philippe.pasero@igh.cnrs.fr (P.P.); 2 Chemical Biology of Cancer Laboratory, CNRS UMR 3666, INSERM U1143, 75005 Paris, France; raphael.rodriguez@curie.fr; 3 Institut Curie, 26 rue d'Ulm, CEDEX 05, 75248 Paris, France; 4 PSL Université, 60 rue Mazarine, 75006 Paris, France; 5 INSERM U1236, University of Rennes 1, Etablissement Français du Sang, 35000 Rennes, France; cogne@unilim.fr; 6 Laboratory for Monitoring Innovative Therapies, Department of Biological Hematology, CHU Montpellier, 34295 Montpellier, France; 7 UFR de Médecine, University of Montpellier, 34003 Montpellier, France; 8 Institut Universitaire de France, 75005 Paris, France

**Keywords:** multiple myeloma, transcription replication conflicts, R-loops, G-quadruplexes, genomic instability, tumorigenesis, plasma cells

## Abstract

**Simple Summary:**

Multiple myeloma is a hematologic cancer characterized by the accumulation of malignant plasma cells in the bone marrow. It remains a mostly incurable disease due to the inability to overcome refractory disease and drug-resistant relapse. Oncogenic transformation of PC in multiple myeloma is thought to occur within the secondary lymphoid organs. However, the precise molecular events leading to myelomagenesis remain obscure. Here, we identified genes involved in the prevention and the resolution of conflicts between the replication and transcription significantly overexpressed during the plasma cell differentiation process and in multiple myeloma cells. We discussed the potential role of these factors in myelomagenesis and myeloma biology. The specific targeting of these factors might constitute a new therapeutic strategy in multiple myeloma.

**Abstract:**

Plasma cells (PCs) have an essential role in humoral immune response by secretion of antibodies, and represent the final stage of B lymphocytes differentiation. During this differentiation, the pre-plasmablastic stage is characterized by highly proliferative cells that start to secrete immunoglobulins (Igs). Thus, replication and transcription must be tightly regulated in these cells to avoid transcription/replication conflicts (TRCs), which could increase replication stress and lead to genomic instability. In this review, we analyzed expression of genes involved in TRCs resolution during B to PC differentiation and identified 41 genes significantly overexpressed in the pre-plasmablastic stage. This illustrates the importance of mechanisms required for adequate processing of TRCs during PCs differentiation. Furthermore, we identified that several of these factors were also found overexpressed in purified PCs from patients with multiple myeloma (MM) compared to normal PCs. Malignant PCs produce high levels of Igs concomitantly with cell cycle deregulation. Therefore, increasing the TRCs occurring in MM cells could represent a potent therapeutic strategy for MM patients. Here, we describe the potential roles of TRCs resolution factors in myelomagenesis and discuss the therapeutic interest of targeting the TRCs resolution machinery in MM.

## 1. Introduction

Human plasma cells (PCs) represent the final stage of B lymphocyte differentiation and play an essential role in the humoral immune response by secreting antibodies. They are mainly located in the bone marrow, where they normally represent only 0.25% of all bone marrow mononuclear cells [1]. In lymph nodes, native B cells are induced to become either memory B cells (MBCs) or plasmablastic cells. In the latter case, plasmablastic cells migrate rapidly to the bone marrow niches or other tissues where they find the adequate microenvironment for long-term survival [2,3]. Mature PCs are characterized by very high immunoglobulin (Ig) secretion.

In adult life, B lymphocytes are continuously produced, and during their differentiation in either MBC or towards PCs, they undergo several genetic rearrangements, associated with DNA breaks to finally assure the immense variability in Igs: VDJ recombination, Ig class switch recombination (CSR), and somatic hyper-mutation (SHM). CSR and SHM take place in the germinal center of the secondary lymphoid organs. These events need to be tightly regulated to ensure an efficient immune response without auto-immune reactions, and to prevent tumorigenesis. This requires adequate processing of the physiological R-loops occurring in guanine-rich switch (S) regions of the immunoglobulin heavy chain (IgH) locus. R-loops are three-stranded nucleic acid structures, formed by the annealing of an RNA moiety with double-stranded DNA constituting an RNA:DNA hybrid [4]. These structures are physiologically enriched near promoters and transcription termination sites, and are involved in CSR, transcription initiation and termination, and telomere elongation [5]. In contrast, unscheduled R-loop formation interferes with replication fork progression and increases the collision rate between the replication and transcription machineries [6], known as transcription/replication conflicts (TRCs) [7]. Therefore, R-loops not only facilitate programmed recombination such as CSR but also represent an important source of spontaneous genomic instability, and their formation must be tightly regulated to prevent tumorigenesis [8,9,10].

**Figure 1:**
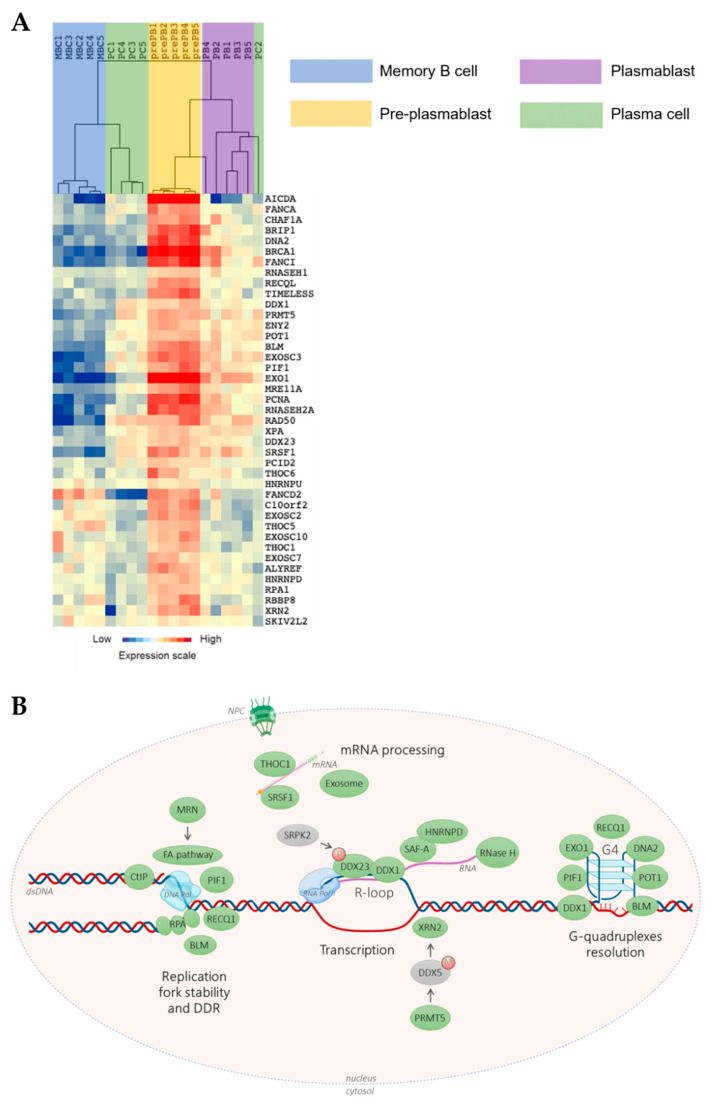
Transcription/replication conflicts (TRCs) resolution genes expression during memory B cells (MBCs) differentiation. (**A**) The genes significantly overexpressed in pre-plasmablasts (prePBs) compared to MBCs, PBs and plasma cells (PCs) were determined with a SAM (significance analysis of microarrays) multiclass analysis (false discovery rate (FDR) = 0), identifying 41 unique genes. When a gene was assayed by several probe sets, the probe set with the highest variance was used. An unsupervised hierarchical clustering was run on this list of 41 unique genes. The normalized expression value for each gene is indicated by a color, with red representing high expression and blue representing low expression. (**B**) TRCs resolution machinery. Green: factor overexpressed in prePBs. Grey: non-overexpressed factor. M: methylation. P: phosphorylation. S: SUMOylation. Simple arrow: activation or recruitment. Double arrow: interaction. dsDNA: double-strand DNA. G4: G-quadruplex. NPC: nuclear pore complex. The scheme was made using BioRender.

Our group developed a multi-step cell culture system to model B-cell to PC differentiation where various combinations of cytokines and activation molecules are needed to reproduce the sequential PC differentiation steps occurring in the different organs/tissue in vivo [11,12,13]. MBCs differentiate into pre-plasmablasts (prePBs), plasmablasts (PBs), early PCs, and finally, long-lived PCs that produce high Ig amounts [11,12,13]. PrePBs have been identified in human lymph nodes, tonsils, and bone marrow [12,14]. This transitional stage is characterized by the absence of both B-cell and PC markers. In particular, at this stage, DNA replication and transcription rates dramatically increase since prePBs start to produce large amounts of Igs and still have a high proliferation rate [12,15], a unique situation possibly prone to give rise to increased TRCs. Therefore, a tight regulation between transcription and replication at this step is critical to avoid unscheduled DNA breaks that might lead to oncogenic transformation. Using our mRNA expression data that are available at ArrayExpress (http://www.ebi.ac.uk/arrayexpress/ accessed on 23 July 2021, E-MTAB-1771, E-MEXP-2360 and E-MEXP-3034), we found that several factors and protein complexes involved in TRC prevention or resolution are overexpressed in prePBs (Figure 1), highlighting the importance of mechanisms required for proper TRC processing during human PC differentiation. Interestingly, several of these factors are overexpressed in malignant PCs from patients with multiple myeloma (MM) compared with PCs from healthy donors. Among these factors, XRN2, DDX1, DDX23 HNRNPU, HNRNPD, SRPK2 and SRSF1 have been identified in the R-loop interactome, reinforcing their role in R-loop biology [16]. Multiple myeloma is an incurable hematological cancer characterized by malignant PC accumulation in the bone marrow. These cells are characterized by high genomic instability, due to oncogene-induced replication stress, and cell cycle deregulation [9,17,18]. Genomic abnormalities in MM involve somatic mutations and translocations between an oncogene (MMSET, CCND3, CCND1, MAF) and the open chromatin of a topologically-associated domain (TAD) of an IgH locus. As mentioned above, since malignant PCs produce high Ig amounts while they keep dividing, an ever increasing efficiency of TRC resolution might confer a significant selective advantage during myelomagenesis and disease progression. Therefore, promoting TRC persistence could constitute an interesting new therapeutic strategy to treat MM.

In this review, we investigated the expression of factors involved in TRC resolution during B-cell to PC differentiation, and discuss their potential roles in myelomagenesis. We also explored the therapeutic interest of strategies to increase TRC occurrence in malignant PCs.

## 2. Management of Transcription/Replication Conflicts (TRCs) in Normal Cells Is Critical to Prevent Genomic Instability

During normal PC differentiation, the prePB stage is associated with a high rate of cell proliferation induced after B-cell activation, with around 50% of cells in S phase. Besides, Ig secretion starts at this stage resulting in strong Ig gene transcription [12,15]. PrePBs are hence exposed to both transcriptional and replication stresses that could enhance the occurrence of DNA lesions due to collisions between replication and transcription machineries. The role of TRCs in cancer has been significantly studied [19,20], however the specific involvement of this process in PC tumorigenesis have never been described. We hypothesized that, particularly at the prePB stage, TRCs have to be carefully managed. First, we sought to identify genes involved in TRC resolution that are significantly overexpressed in prePBs during B-cell to PC differentiation. By reviewing the existing literature, we identified 83 genes involved in TRC resolution (Table S1). Then, we quantified their expression during PC differentiation using our in vitro model and Affymetrix microarray data (data are available at ArrayExpress; http://www.ebi.ac.uk/arrayexpress/ accessed on 23 July 2021, E-MTAB-1771, E-MEXP-2360 and E-MEXP-3034). GenomicScape webtool [21] and multi-class SAM (significance analysis of microarrays) analysis revealed that 41 of these genes were significantly overexpressed in prePBs compared with MBCs, PBs and PCs, with a false discovery rate <5% (Figure 1 and Table S2). Among them, we focused on 38 genes that are described hereafter.

### 2.1. R-Loop Resolution Genes

#### 2.1.1. RNase H1/2, Replication Protein A (RPA)

Ribonucleases H (RNase H) comprise a group of enzymes that degrade the RNA strand of RNA:DNA hybrids. There are two main classes of RNases H in eukaryotic cells, distinguished by their primary sequences and substrate specificities [22,23]. RNase H1 is a monomeric enzyme that resolves long RNA:DNA hybrids and removes the ribonucleotides in DNA that are at least four nucleotides long [22]. In mammalian cells, RNase H1 is essential for R-loop processing during mitochondrial DNA replication and its deletion causes embryonic lethality in mice [24,25,26]. RNase H1 depletion also resulted in increased nuclear RNA:DNA hybrids, DNA damage and slowing of DNA replication forks [27], implying that RNase H1 plays an important role in resolving TRCs in the nucleus. 

RNase H2 is a heterotrimeric complex that processes hybrids and removes single ribonucleotide in DNA. Ribonucleotides are incorporated during genome replication at a remarkably high rate [28,29]. These mis-incorporated nucleotides in nascent DNA need to be efficiently removed by the ribonucleotide excision repair (RER) pathway, a process similar to Okazaki fragment processing after incision by RNase H2 [30]. Biallelic mutations in RNase H2 are linked to a neuroinflammatory disease, Aicardi-Goutières syndrome, presumably through the accumulation of cytosolic DNA fragments and the activation of the DNA sensing cGAS/STING pathway [31,32]. RNase H2 plays, therefore, a critical role in the maintenance of genome integrity. During normal PC differentiation, the prePB stage is associated with high cell proliferation following B-cell activation with about half the cell population in the S phase. Increased expression of RNase H2 in prePBs could be critical to reduce R-loop levels and maintain genome integrity (Figure 1A).

RPA is a major single-stranded DNA (ssDNA) binding protein conserved in all eukaryotes [33,34]. It has been shown that RPA colocalizes with R-loops. In vitro, RPA stimulates RNase H1 activity on R-loops. Moreover, RPA-RNase H1 interaction is critical for RNase H1 binding to R-loops [35]. Importantly, RPA interacts with various TRC resolution factors that will be described below and, therefore, is a key regulator of TRC-induced genomic instability. As RPA is involved also in replication fork progression and restart, its function is not restricted to prevent unscheduled R-loop formation [36]. Importantly, it has been shown that the proteasome inhibitor Bortezomib, used in the treatment of MM, prevents DNA resection and thus RPA recruitment onto ssDNA [37,38]. Moreover, higher *RPA* expression is associated with an increased bone marrow infiltration of MM cells which is associated with a poor outcome [39].

#### 2.1.2. The DEAD-Box Protein Family of Helicases

The DEAD-box RNA helicase 1 (DDX1) is involved in RNA metabolism [40,41] and DNA double strand break (DSB) repair [42,43]. At DSB sites, DDX1 forms foci to resolve DNA:RNA hybrids [42,43], and could also be involved in G-quadruplex (G4) structures remodeling [44]. G4s are four-stranded secondary DNA structures, constituted of at least two stacked guanine tetrads stabilized by Hoogsteen hydrogen bonds and cations [45,46]. These highly stable non-canonical structures are present at telomeres, at the promoter of many genes, and at origins replication [47,48]. A link between G4 structures and R-loops has been proposed since they are both promoted by Guanine-richness (G-richness), and G4s can form in the displaced DNA strand of a R-loop and stabilize it [49,50]. Moreover, a recent study demonstrated that in human cells, genomic instability caused by G4 stabilizers is mediated by R-loop formation [51], reinforcing the interplay between these structures. Interestingly, DDX1 could promote CSR by a mechanism involving G4 structures conversion into R-loops [44]. Long non-coding RNAs (lncRNAs) are transcribed from the intronic S region of the IgH locus (called germline transcripts), and are involved in the formation of co-transcriptional R-loops during CSR that allows the activity of the key CSR enzyme, namely Activation-Induced cytidine Deaminase (AID) [52,53]. Ribeiro de Almeida et al. described a post-transcriptional mechanism by which G4s form in these lncRNAs after their splicing and are subsequently recognize by both AID and DDX1. The latter would promote the conversion of G4 RNAs into RNA:DNA hybrids at the S region, thereby allowing AID targeting and activity. The authors showed that CSR is impaired in *Ddx1* knock-out mice, and DDX1 depletion reduces R-loop levels at IgH S regions [44].

Another DEAD-box RNA helicase, DDX23, is part of the U5 spliceosomal ribonucleoprotein particle (U5 snRNP) involved in messenger RNA (mRNA) splicing, depending on its phosphorylation by SRPK2 [54]. A recent study proposed that the accumulation of R-loops induces RNA polymerase II (RNA Pol II) pausing which will recruit DDX23 to resolve these R-loops [55]. This process requires DDX23 phosphorylation by SRPK2. Upon depletion of both factors, R-loop levels and genomic instability are enhanced.

### 2.2. mRNA Maturation

#### 2.2.1. Serine/Arginine Splicing Factor 1 (SRSF1)

SRSF1 (or ASF/SF2) is an mRNA splicing factor that belongs to the serine/arginine-rich family [56,57,58]. SRSF1-depleted cells are hyper-mutagenic, with DNA rearrangements and increased DSB formation, and their genetic instability is R-loop-dependent. In vitro, SRSF1 associates with RNA Pol II to prevent R-loop formation, and it has recently been shown that SRSF1 depletion in human cells increases R-loop levels [59,60]. Moreover, SRSF1 is required for the SUMOylation of Topoisomerase I (Top1) [61], an enzyme that relieves torsional stress during transcription and replication [62,63]. The SUMOylation is a post-translational modification performed by a member of the Small Ubiquitin-Like Modifier (SUMO) family such as SUMO1. This modification allows the interaction of Top1 with RNA Pol II at actively transcribed genes, and the recruitment of mRNA processing factors preventing R-loop formation. Importantly, the authors showed that Top1 SUMOylation reduces its catalytic activity meaning that the prevention of R-loops might not involve supercoiled DNA relaxation. Instead, they hypothesized that this inhibition could prevent the formation of trapped Top1-DNA complexes that create genomic instability [61,64].

#### 2.2.2. Heterogeneous Nuclear Ribonucleoprotein U (HNRNPU) and D0 (HNRNPD)

HNRNPU (or SAF-A) is an RNA processing factor involved in transcription elongation, RNA stability, and splicing [65,66,67]. HNRNPU is transiently recruited to DNA damaged sites and then rapidly released from chromatin, suggesting a role in DNA repair [68]. Its release depends on transcription and on the activity of the three PI3-kinase-related protein kinase (PIKK) controlling the DNA damage response (DDR), namely ATM, ATR and DNA PK [69]. Upon inhibition of these kinases, HNRNPU remains on chromatin, and R-loop levels are increased, suggesting that its release is important for R-loop resolution at DNA damaged sites.

A recent study showed that HNRNPD interacts with HNRNPU [70]. HNRNPD regulates DDR genes and acts to preserve genomic integrity [71]. It was shown that HNRNPD is necessary for proper DNA end resection during homologous recombination and for the removal of R-loops [70]. HNRNPD depletion reduces HNRNPU recruitment to damaged sites and induces R-loop accumulation. Importantly, a study showed that ILF2 is critical in the pathophysiology of MM cells with 1q21 amplification. They showed that ILF2 interacts with HNRNP U/D among other RNA-binding proteins and modulates the splicing of DNA repair genes [72].

Interestingly, FANCD2 recruits HNRNPU and the DDX47 helicase under replication stress, and HNRNPU or DDX47 depletion leads to R-loop accumulation [73].

#### 2.2.3. The THO/TREX Complex and TREX2

The THO-TREX complex participates in messenger ribonucleoprotein (mRNP) biogenesis and mRNA export [74,75]. This complex has also been involved in preventing transcription-associated genomic instability [76]. Indeed, yeast THO mutants display growth defects, and require a functional S-phase checkpoint for survival upon R-loop accumulation [77]. In these mutants, transcriptional downregulation is observed genome-wide, but particularly at long, highly expressed, and G-rich genes. Interestingly, the recruitment of Rrm3 (a PIF-family helicase that promotes replication progression across DNA obstacles) to transcribed genes is enhanced in THO-depleted mutants, and this recruitment is RNase H-sensitive [78]. Gomez-Gonzalez et al. thus propose that THO acts genome-wide to prevent transcription-dependent replication defects related to R-loop formation.

In human cells, THO depletion impairs transcription elongation, increases DNA damage and spontaneous recombination in a RNase H-sensitive manner [79]. Of note, AID overexpression in THO-depleted murine B cells increases genomic instability and CSR. Indeed, AID catalyzes the deamination of a cytosine in the displaced DNA strand of a R-loop, that will be recognized and processed by DNA repair mechanisms leading to DNA breaks. Replication is altered in THO-depleted cells and the authors discussed the possibility that defects in replication termination or inactivation of DNA damage checkpoints would lead to longer replication tracks. They also hypothesized that the impairment of transcription elongation in those cells would reduce transcription activity and promote the progression of the replication fork. Moreover, THO associates with the DEAD-box RNA helicase UAP56 (DDX39B) that was shown to prevent R-loop-mediated genomic instability and unwind R-loops in vitro [75,80].

Interestingly, a recent paper showed that the Sin3A histone deacetylase (HDAC) interacts with THOC1, a member of the THO complex [81]. The yeast homolog of Sin3A suppresses R-loop-dependent genomic instability, and in human cells genomic instability induced by Sin3A depletion can be rescued by RNase H-overexpression. In Sin3A-depleted cells as well as in THOC1-depleted cells, histone acetylation is enhanced and upon incubation with a HDAC inhibitor, R-loop levels are increased. It is most likely that chromatin opening due to increased acetylation facilitates R-loop formation. This can also explain why in THO-depleted cells, replication forks are faster, but fork pausing or stalling occurs more frequently [81]. Finally, a recent study showed that THOC1 depletion in hepatocellular carcinoma cells induces R-loop accumulation and increases sensitivity to cisplatin [82].

The THSC-TREX2 complex is involved in mRNA export, interacts with the nuclear pore complex [83,84,85], and has a role in alleviating transcription-associated DNA damage. TREX2 yeast mutants display high level of transcription-associated hyperrecombination and downregulation of long, highly transcribed, and G-rich genes, as observed in THO mutants. The two main components of the TREX2 complex, Thp1 (PCID2) and Sac3 (GANP), act as a complex and bind to highly transcribed genomic regions to which THO also binds. Moreover, in yeast TREX2 mutants, TRCs are increased and replication forks stall at Sac3 and Thp1 binding sites. However, the direct involvement of R-loops in this process remains elusive [86]. Another study on TREX2 role in TRC resolution in human cells [87] found that TREX2 depletion increases genomic instability and DNA damage. Of note, the homologous recombination factor BRCA2 interacts with two TREX2 components, PCID2 and DSS1, and upon BRCA2 depletion the accumulation of R-loops is observed [87]. However, the precise role of TREX2 in TRC and R-loop resolution is still unclear.

### 2.3. RNA Processing and Degradation

#### 2.3.1. The RNA Exosome

The RNA exosome is a ribonucleolytic complex involved in RNA processing and degradation [88,89], and is composed of nine non-catalytic subunits (EXOSC1-EXOSC9) and two catalytic subunits (EXOSC10 and DIS3). DIS3 mutations are involved in MM progression [90]. Indeed, DIS3 mutations are associated with a poor prognosis and are associated with significant transcriptional changes [91,92]. Moreover, germline variants in *DIS3* were identified in familial MM [93]. A recent study demonstrated that EXOSC10 degrades DNA damage-induced long non-coding RNAs that are synthesized at DSB sites and might be involved in R-loop formation [94]. Moreover, the RNA exosome cooperates with the helicase Senataxin (SETX) for R-loop removal, and EXOSC9 colocalizes with SETX in an R-loop-dependent manner [95]. SETX or RNA exosome depletion in B cells increases genomic instability and impairs CSR [96,97]. SETX could resolve R-loops and recruit the RNA exosome for RNA degradation. Optimal activity of the RNA exosome complex has been shown to be mandatory for clearing non-coding RNAs from R-loops formed at S regions, thus facilitating the occurrence of cytidine deamination by AID on both strands of R-loops for optimal CSR [98]. The specific catabolism of non-coding RNAs by the DIS3 subunit was additionally shown to impact TAD structures genome-wide [99].

#### 2.3.2. 5′-3′ Exoribonuclease 2 (XRN2)

XRN2 is involved in transcription termination [100,101]. XRN2 could link transcription and DNA repair because upon DNA damage, it forms foci that colocalize with several DDR factors. Of note, XRN2 colocalizes with R-loops upon UV exposure in a transcription-dependent manner, and XRN2 depletion increases R-loop and DSB levels [102]. It has been shown recently that XRN2 resolves RNA:DNA hybrids to allow the initiation of DNA repair by non-homologous end-joining (NHEJ) [103]. XRN2 could work together with several factors for R-loop resolution. For example, the R-loop unwinding activity of SETX allows XRN2 access to RNA in order to degrade it [104]. Moreover, XRN2 interacts with the DDX5 helicase [105,106], and this interaction requires DDX5 arginine methylation by PRMT5 [107]. One hypothesis is that DDX5 unwinds RNA-DNA hybrids and that subsequently XRN2 degrades the RNA moiety. Upon depletion of PRMT5, DDX5, or XRN2, R-loops accumulate at highly transcribed genes [108]. Interestingly, high PRMT5 expression is associated with an adverse outcome in MM [109]. Moreover, PRMT5 depletion or inhibition in MM cells inhibits cell growth and induce apoptosis in association with NFκB pathway downregulation [109].

### 2.4. Fork Protection and Stability

#### 2.4.1. The Fanconi Anemia Pathway and the MRN Complex

Fanconi anemia (FA) is a rare genetic disorder characterized by congenital abnormalities as well as an increased susceptibility to cancers and to hematopoietic failure [110,111,112]. The best characterized function of the FA pathway is the removal of inter-strand crosslinks and subsequent DNA repair by HR [113,114]. The FA pathway has also been implicated in R-loop resolution. Specifically, two studies demonstrated that FANCD2 is required for efficient R-loop resolution the prevention of genomic instability [115,116]. Interestingly, high levels of FANCD2 expression are associated with shorter survival in MM [117]. Upon FANCD2 depletion, DSBs accumulate at R-loop sites in a transcription- and R-loop-dependent manner. When DNA replication is inhibited using aphidicolin, a DNA polymerase inhibitor, FANCD2 accumulates at large transcribed genes and colocalizes with R-loops in a transcription-dependent manner [115,116]. Another study demonstrated that FANCD2 participates in R-loop elimination at common fragile sites to allow their replication. FANCD2 depletion increases the R-loop-dependent genomic instability at these sites [118]. Monoubiquitylation of the FANCI-FANCD2 complex is required for R-loop resolution [119], and FANCI-FANCD2 binding to the displaced DNA strand or to the RNA tail of the R-loop stimulates its monoubiquitylation. FANCD2 recruitment to chromatin is promoted by transcription R-loop and DNA damage [119]. In FA-deficient cells, R-loops accumulate at some loci to which the FA core complex protein FANCA binds, and FANCD2 foci are sensitive to RNase H1 treatment [115]. Moreover, the DEAD/DEAH helicase FANCM might participate in RNA:DNA hybrid unwinding, at least in vitro, and its depletion induces R-loop accumulation [116]. Therefore, the FA pathway plays a significant role in R-loop resolution and in genomic instability prevention, through several effectors. Interestingly, FANCI is part of a gene prognostic signature in MM patients [120]. FANCA depletion has been identified in a CRISPR-Cas9 screen to sensitize MM cells to melphalan [121].

A recent study also demonstrated that the MRN (MRE11, RAD50, NBS1/NBN) complex has a role in promoting R-loop resolution by the FA pathway [122], and that this role is independent from its catalytic activity. The authors showed that the MRN complex is required for FANCD2 and FANCM recruitment to R-loops. In MRN-depleted cells, R-loop accumulation and R-loop-dependent DNA damage are increased. MRE11 and RAD50 expression levels correlate with high bone marrow infiltration in MM [39].

The FA pathway could also contribute to G4 resolution. Indeed, the DNA helicase FANCJ (BRIP1) can bind to and unwind G4 structures in vitro [123,124,125]. FANCJ depletion increases sensitivity to the G4 stabilizing molecule telomestatin suggesting a role in preventing G4-induced genomic instability [123]. Moreover, cells derived from patients with FA and FANCJ deficiency accumulate large genomic deletions in G4-prone regions, reinforcing the protective role of FANCJ against DNA damage induced by G4 structures [124]. In *Xenopus laevis* egg extracts, treatment with G4 stabilizers promotes RNA Pol II stalling and increases the FANCJ requirement to ensure the efficient replication of G4-containing regions [126]. Accordingly, FANCJ bypasses G4 structures in vitro and unwinds downstream DNA to allow its faithful replication [125]. Importantly, cancer-associated FANCJ mutations [127] reduce its ability to unwind G4 structures and increase sensitivity to G4 stabilizing agents [128].

Finally, a recent study reported that FANCJ interacts with the helicase REV1 via a PCNA-interaction peptide (PIP)-like motif to form a G4-resolving complex [129]. REV1 binds preferentially to G4 DNA substrates in vitro, prevents G4 folding, and disrupts G4 DNA structures [130]. Moreover, its depletion could increase the mutational rate at G4 DNA sites [131].

#### 2.4.2. Breast Cancer Susceptibility Gene 1 and 2 (BRCA1 and BRCA2)

BRCA1 and BRCA2 are part of the FA pathway [132]. Specifically, BRCA1 is a tumor suppressor with an extensively documented role in DNA repair and homologous recombination (HR) [133]. In MM, the FA/BRCA pathway contributes to melphalan resistance and targeting this pathway can potentiate the response to melphalan treatment [134,135]. Importantly, the NFκB pathway frequently deregulated in MM is known to promote HR through stimulation of BRCA1 and CtIP [136,137]. BRCA1 is recruited to R-loops and forms a complex with the SETX helicase to suppress co-transcriptional and R-loop-induced DNA damage. Moreover, BRCA1 is associated with transcription termination sites of highly transcribed genes that are enriched in genomic alterations in BRCA1-deficent breast tumors [138]. Of note, R-loop accumulation is observed upon *BRCA1* knock-down in human cells [87,139] and in BRCA1 mutation-carrying precancerous breast tissue [140]. Moreover, R-loops preferentially accumulate at loci associated with RNA Pol II pausing in these cells [140].

A recent study showed that BRCA2 associates with the DEAD-box RNA helicase DDX5, which resolves RNA:DNA hybrids at DSBs to facilitate DNA repair [105,106,141,142]. Their interaction is enhanced upon DNA damage and reduced by RNase H1 overexpression. BRCA2 depletion leads to R-loop accumulation at DSBs and promotes DDX5 recruitment to DNA damage sites, presumably to facilitate homologous recombination [141]. Moreover, BRCA2 can stimulate DDX5 unwinding activity in vitro [141]. Importantly, DDX5 interaction with the BRCA2 mutant T207A, found in breast cancer cells from patients, is reduced compared to wild-type BRCA2, and R-loops levels are increased in cells that overexpress this variant [141].

#### 2.4.3. Proliferating Cell Nuclear Antigen (PCNA) SUMOylation

PCNA, one of the main components of the replication fork, enhances DNA polymerase processivity and is involved in DDR and genome stability maintenance [143]. In MM, PCNA expression increases with disease progression [144,145]. PCNA targeting induces apoptosis and increases the efficacy of several treatments including melphalan, doxorubicine, thalidomide and azacitidine [146]. PCNA can be conjugated to SUMO1 to prevent homologous recombination at DSBs [147,148], and conjugation with SUMO2 has recently been involved in TRC resolution [149]. Indeed, PCNA conjugation to SUMO2 on transcribed genes during S phase positively regulates replication fork progression, in a RECQ5-dependent manner. This conjugation destabilizes RNA Pol II binding, thus reducing transcription and facilitating replication [149]. Interestingly, SUMO2-conjugated PCNA interacts with the histone chaperone CAF1, and enhances CAF1-dependent histone deposition, thereby forming repressive chromatin [149]. Upon SUMO2-PCNA conjugation abrogation, TRCs and DNA damage are increased. The helicase RECQ5 allows the interaction between PCNA and RNA Pol II, and between PCNA and SUMO2, and suppresses TRC-induced DSBs through chromatin remodeling [149]. Interestingly, SUMO2 could be involved in induction of bortezomib resistance upon silencing of Sentrin/SUMO-specific proteases-2 (SENP2) [150].

#### 2.4.4. CtBP-Interacting Protein (CtIP)

CtIP (RBBP8) is a 5′ flap endonuclease a regulator of MRN activity, critical for DNA end resection at DSBs [151,152,153]. CtIP expression levels are associated with relapse and with a poor prognosis in MM [154]. A recent study showed a role for CtIP in R-loop processing [155]. The authors described a mechanism by which CtIP would recognize and process 5′ flaps that are present inside the R-loop structure, to promote the activity of helicases that would remove the RNA strand and therefore resolve the R-loop structure. Accordingly, CtIP-depleted cells show more R-loops but fewer DNA breaks. CtIP depletion in both human and yeast cells reduces their survival upon exposition to the Top1 inhibitor camptothecin (CPT), and increases R-loop formation in a transcription-dependent manner. Moreover, in CtIP-depleted cells exposed to CPT, transcription patterns are altered mainly for R-loop-prone genes, and SETX chromatin binding is increased. R-loop resolution by CtIP requires its nuclease activity and is observed in both untreated and CPT-treated cells. Intriguingly, concomitant loss of CtIP and of the XPG nucleotide excision repair endonuclease abrogates R-loop formation, whereas depletion of each protein on its own promotes R-loop formation. In yeast, depletion of the CtIP homolog Sae2 increases RNA Pol II stalling upon CPT exposure specifically during S-phase [155].

#### 2.4.5. Exonuclease 1 (EXO1)

EXO1 is a 5′-3′ exonuclease involved in mismatch repair and DSB resection during homologous recombination [156]. EXO1 depletion in human cells leads to spontaneous telomere defects, to the stalling of replication forks preferentially at G4 structures and enhances the cell sensitivity to G4 stabilizers. EXO1 could have a protective role on the replication fork that stall in front of a G4 structure, by resecting the nascent DNA and promoting repair by homologous recombination. Accordingly, EXO1-depleted cells display less resection around G4 structures and the collapsed forks are mainly repaired by the error-prone NHEJ repair pathway [157].

#### 2.4.6. Transcription Coupled Nucleotide Excision Repair (TC-NER) Exonucleases

Structure-specific endonucleases, such as XPF and XPG, recognize and process specific secondary DNA structures to facilitate replication or DNA repair [158]. As mentioned before, it is thought that R-loop structures contain at least two 5′ flaps that could be recognized by such enzymes [155]. XPF and XPG process R-loop structures formed in the switch region of the IgH locus in vitro [159]. In human cells, XPG and XPF can process R-loops induced by depletion of the RNA processing factor Aquarius (AQR), in a nuclease activity-dependent manner [160]. Moreover, XPG cleaves R-loops that accumulate upon depletion of various R-loop resolution factors (AQR, SETX, or SRSF1) or upon CPT exposure. This mechanism requires other components of the transcription-coupled nucleotide excision repair (TC-NER) such as XPA, XPB, XPD and CSB. XPF can bind to R-loops and is enriched at R-loop sites of gene bodies upon cell exposure with CPT, and can induce ssDNA breaks within R-loops [161]. Moreover, XPG activity is necessary for RAD52-dependent R-loop resolution [162]. It has been hypothesized that the ability of TC-NER components to process R-loops could be a way to distinguish between physiological and unscheduled R-loops, by acting only on gene bodies R-loops that are detrimental for the replication fork progression [163]. In MM, shorter overall survival is associated with single nucleotide polymorphisms (SNPs) in XPG and XPA genes [164]. Additionally, adult T-cell leukemia cells accumulate R-loops and often lack TC-NER factors, such as XPF and XPG [165].

### 2.5. G-Quadruplexes Resolution

#### 2.5.1. The RecQ family of Helicases 

Bloom’s syndrome helicase (BLM) is a DNA helicase of the RecQ family [166] involved in replication fork restart, notably via G4 unwinding [167,168,169]. BLM-depleted cells are characterized by increased genomic instability, DNA damage, sister chromatid exchanges (SCE) and micronuclei formation [170,171]. Upon BLM depletion, genes enriched in putative G4 sequences are downregulated, suggesting that the G4 unwinding activity of BLM plays an additional role in transcription regulation [172,173]. Moreover, in BLM-depleted cells, SCEs occur mostly at G4 motif-containing sites in actively transcribed genes [171]. Interestingly, the phenotype of BLM-depleted cells can be reversed by RNase H overexpression, suggesting that R-loops are involved in the phenotypes of BLM deficiency. BLM colocalizes with R-loops, and its depletion leads to R-loop accumulation [170]. Sgs1, the yeast homolog of BLM, can reduce R-loop formation and DNA damage levels at fragile sites and R-loop-prone genomic loci. Sgs1-depleted cells are characterized by increased R-loop levels, transcription-associated recombination, and DNA damage. This phenotype is exacerbated when Sgs1 loss is combined with the loss of another TRC resolution gene (TREX, RNase H, or the Senataxin homolog Sen1) [170]. Altogether, these data show that BLM is a crucial helicase that can act on both G4s and R-loops to reduce DNA damage formation.

Moreover, the HERC2 E3 ligase can enhance BLM unwinding activity, presumably through promoting the interaction of BLM with RPA. HERC2 promotes this interaction mainly during S phase and its E3 ligase activity is required for BLM helicase activity. This could involve HERC2-mediated phosphorylation and ubiquitination of RPA. HERC2-depleted cells display increased G4 formation and SCEs occurrence. They are sensitized to G4 stabilizing molecules, such as pyridostatin and telomestatin [174,175]. Finally, HERC2 E3 ligase activity has an epistatic relationship with RPA regarding G4 resolution [176]. Therefore, HERC2 is an important regulator of G4 resolution.

Additionally, a study demonstrated the direct interaction between Top1 and BLM. It was shown in vitro that Top1 stimulates BLM unwinding activity on RNA:DNA hybrids, and reciprocally, BLM stimulates Top1 activity on DNA:RNA hybrids, in a helicase activity-independent manner [177]. More recently, RECQ DNA helicase BLM was shown to be overexpressed in prePBs during B to PC differentiation [178]. BLM could restrain the deleterious consequences of R-loop mediated replication stress in highly proliferative prePB cells upon activation of transcription. In the absence of BLM, these cells would, therefore, accumulate stalled forks leading to chromosome breaks due to their inability to efficiently remove R-loops and G4 structures [178].

RECQ1 is an ATP-dependent DNA helicase that has a role in genome maintenance and DDR. Its depletion increases chromosomal breaks and sensitizes cells to replication blocking agents [179,180]. Like BLM, RECQ1 helicase activity is also stimulated by RPA [181]. Moreover, in MM cells, RECQ1 depletion induces and accumulation of cells in G1 and G2/M phases and increased apoptosis, whereas RECQ1 overexpression protects these cells from bortezomib or melphalan-induced cell death [182]. Melphalan is an alkylating agent classically used in the treatment of MM. Moreover, RECQ1 mRNA levels are upregulated upon DNA damage in a p53-dependent manner [183]. Interestingly, genes downregulated upon RECQ1 depletion are enriched in G4 motifs, and RECQ1 can bind to G4 structures at their promoter [184,185]. Of note, RECQ1 interacts with several members of the TREX1 complex, suggesting that it could cooperate with this complex in TRC resolution [182]. However, RECQ1 cannot unwind G4 structures on its own in vitro [168]. Though, RECQ1 might contribute to G4 motif-related DNA repair, since RECQ1 is rapidly recruited to oxidized chromatin. Additionally, it has been shown that the guanine residues present in G4 motif-containing promoters are prone to oxidization, forming an 8-oxoguanine lesion [186]. In line with this hypothesis, a recent study showed that PARP1, a sensor of DNA damage, is recruited to oxidized G4s and that this interaction can promote PARP1 activation. This could lead to G4s sensing and signaling to recruit DNA repair enzymes for G4 removal [187]. RECQ1 interacts with PARP1and its depletion re-sensitizes MM cells to PARP inhibitors [182], reinforcing the hypothesis of RECQ1 being involved in G4-related DNA repair. However, RECQ1 role in G4 resolution needs to be clarified.

#### 2.5.2. PIF1

The DNA helicases of the PIF1 family are conserved among eukaryotes. This family comprises two members in budding yeast, Pif1 and Rrm3, whereas in most eukaryotes only PIF1 is present [188]. PIF1 regulates telomerase activity and Okazaki fragment maturation [188]. PIF1 can unwind G4s in vitro [189,190,191]. In budding yeast, it is necessary for the efficient replication of G4-containing sequences [192,193,194]. In the absence of Pif1, replication fork progression is slower. Moreover, Pif1 interaction with PCNA is crucial to allow G4-forming sequences to be replicated [192]. PIF1 and RPA have complementary roles in DNA replication across G4 sequences [195] in vitro, with RPA allowing replication through G4 DNA, and PIF1 unwinding these structures. Their interaction is DNA-dependent [196]. RPA unwinds G4s in vitro [197,198], prevents genomic instability at G-rich motifs, and is crucial for G4 removal [196,199]. Human and yeast RPA prevents G4 formation in telomeres to allow telomere elongation [200].

Interestingly, Pif1 preferentially unwind RNA:DNA hybrids rather than DNA duplexes [201]. Moreover, Pif1 has a role in promoting replication through transfer RNA genes (tDNAs). These structures are enriched in R-loops, and the association of Pif1 with tDNAs is enhanced by RNase H depletion [202]. Therefore, Pif1 could have an additional role in R-loop resolution.

#### 2.5.3. DNA2 and POT1 Roles at Telomeres

DNA2 is a helicase/nuclease involved in the maintenance of genomic stability [203]. Interestingly, it could have a redundant role with the Pif1 helicase at telomeres [204]. Indeed, yeast and human DNA2 bind to telomeric G4 DNA and can cleave these structures in an RPA-dependent manner [205,206]. DNA2 deficiency induces strong telomeric DNA damage [206]. Importantly, DNA2 is found throughout replicating DNA during S phase, but not on telomeres [207], and DNA2-depleted cells are characterized by high genomic instability (chromosome bridges, aneuploidy, replication defects) [208,209]. Moreover, DNA2 reduces replication stress in cancer cells [209]. Mouse *DNA2* homozygous knock-out is embryonic lethal, demonstrating an essential role of this protein [206]. Interestingly, DNA2 interacts with PCNA and FANCD2 that are both involved in TRCs resolution (see above). It stimulates BLM DNA unwinding activity [210], but it is not known whether it also stimulates G4 unwinding.

POT1 is a component of the shelterin complex in charge of telomere protection and binds to the G-rich ssDNA 3′ overhang in telomeres [211]. In vitro, POT1 disrupts G4 formation at telomeres to allow proper elongation by telomerase [212]. The two G4 stabilizing molecules telomestatin and pyridostatin inhibits POT1 binding to telomeres in vitro and in human cells [175,213,214]. POT1 unfolds G4 DNA to produce ssDNA-POT1 complexes [215,216]. However, in ciliates, the POT1 homolog TEBPα (Telomere end-binding protein alpha) participate in the formation of telomeric G4s [217]. Interestingly, POT1 expression is associated with clinical characteristics related to adverse outcome in MM [218].

Despite the lack of studies involving all these factors specifically in malignant transformation of PCs, we hypothesized that the significant overexpression of TRCs resolution genes in prePBs might play an important role during memory B-cell to PC differentiation by preventing genomic instability and thus tumorigenesis. Under normal conditions, only a limited number of memory B cells are able to form new germinal center (GC) after reactivation. During early tumorigenesis, founder mutations acquired by memory B cells as SHM off-targets or resulting from DNA replication errors following B-cell activation can jeopardize this mechanism and yield a set of aberrant memory B cells that progressively outcompete wild-type memory B and naïve B cells along their clonal expansion [219]. Furthermore, participation in successive GC reactions is predicted to result in cumulative acquisition of further off-target mutations in these cells [219]. A recent study investigating the chronological activity of mutational signatures in MM, using a large cohort of 89 whole genome and 973 exome data, estimated that the transformation of a GC B cell occurred during the first second or third decades of life [220]. Furthermore, their data indicated that AID activity is not restricted to the first GC reaction but persists in at least a subset of patients, potentially affecting disease evolution. This supports that pre-malignant MM cells have re-entered the GC for clonal expansion decades before MM diagnosis [220]. Additional levels of genomic instability, related to AID-independent processes are also likely and could be related to replicative stress. Since pre-plasmablastic stage is characterized by high proliferation (50% of cells in S phase) and the beginning of Ig secretion [12], conflicts between transcription (likely mainly at the Ig loci) and replication during PC differentiation impact on replication stress and mutagenesis and might be involved in myelomagenesis. However, the presence of R-loops and G-quadruplexes at myeloma-associated mutation hotspots in prePBs remains to be demonstrated together with the mechanisms protecting them from genomic instability.

## 3. The Potential Role of TRC in Multiple Myeloma Pathophysiology

**Figure 2:**
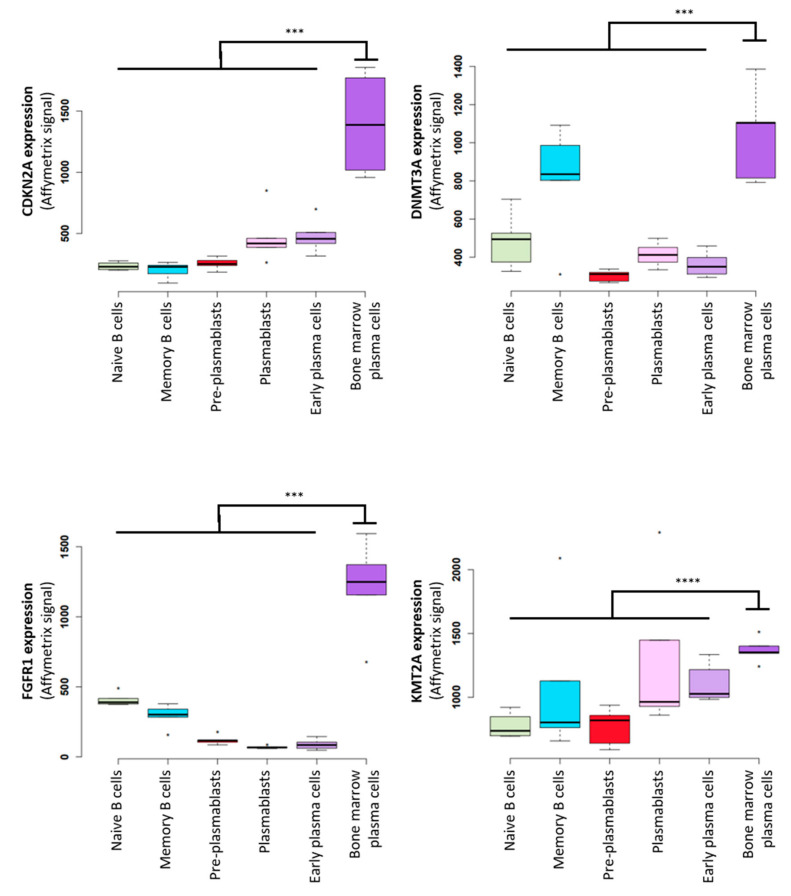
Frequently mutated genes in multiple myeloma (MM) are overexpressed in normal plasma cells. Expression of 4 genes associated with recurrent mutation in MM during B to PC differentiation. Affymetrix microarrays expression box-plots were defined using GenomicScape web tool (http://www.genomicscape.com accessed on 23 July 2021). * indicates a significant difference using Student’s *t*-test (*p* ≤ 0.05), *** *p* ≤ 0.005, **** *p* ≤ 0.0005.

**Figure 3:**
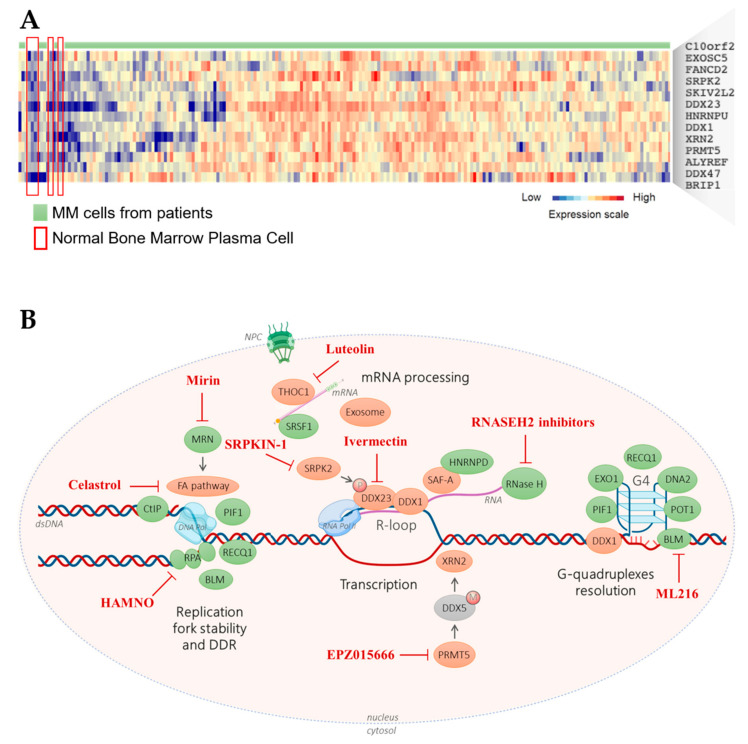
Gene expression profile of TRCs resolution genes in purified MM cells from patients. (**A**) Cluster analysis of TRCs gene expression in normal bone marrow plasma cells (BMPCs, *n* = 5) and purified MM cells from patient (*n* = 206). (**B**) Potent roles of TRCs resolution factors in MM and their inhibitors. Cf legend in Figure 1B. Orange: genes overexpressed in MM patients.

**Figure 4:**
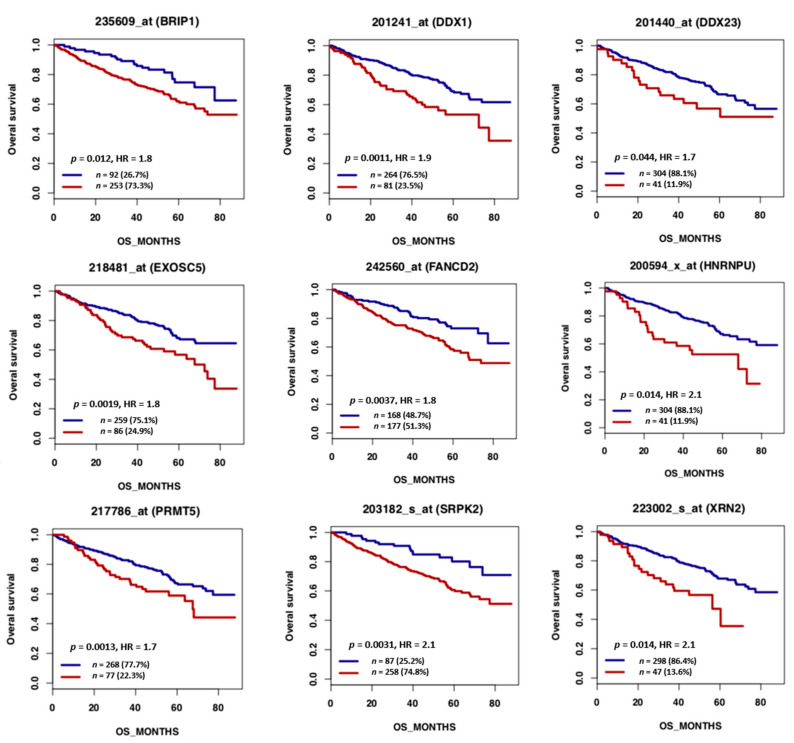
High expression of 9 TRCs resolution genes is associated with a poor outcome in MM patients. Patients of the TT2 cohort (Arkansas, newly diagnosed MM patients treated by High-dose therapy (HDT) and autologous stem cell transplantation (ASCT), *n* = 345) were ranked according to expression of the TRCs resolution genes and a maximum difference in OS was obtained using the Maxstat R function. Red: high expression. Blue: low expression. Kaplan–Meier curves were defined using GenomicScape web tool (http://www.genomicscape.com accessed on 23 July 2021). HR: hazard ratio.

**Figure 5:**
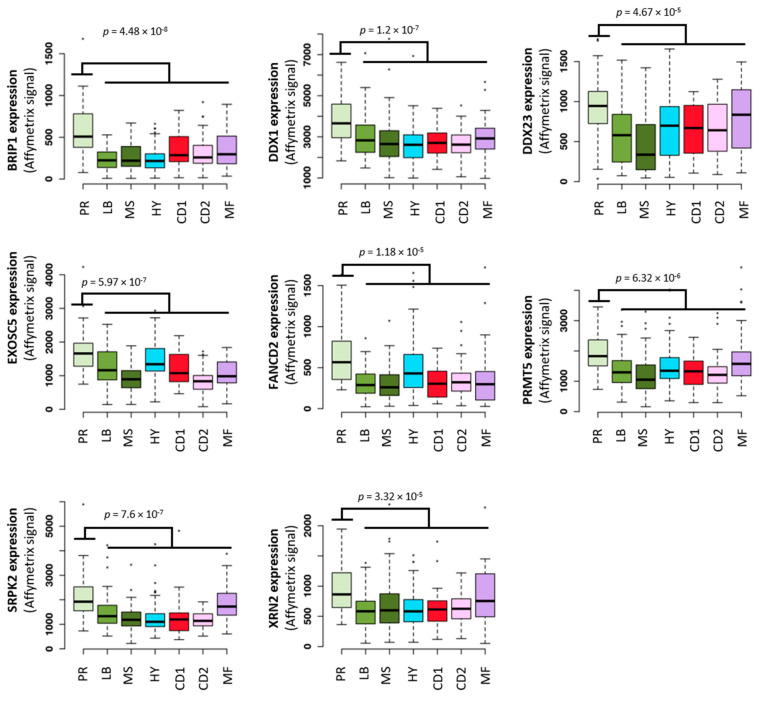
8 TRCs resolution genes are significantly overexpressed in the proliferation molecular subgroup in MM. Expression of 8 TRCs resolution genes in patients of the 7 MM molecular subgroups of the TT2 cohort (Arkansas, newly diagnosed MM patients treated by High dose therapy (HDT) and autologous stem cell transplantation (ASCT), *n* = 345). Affymetrix microarrays expression box-plots were defined using GenomicScape web tool (http://www.genomicscape.com accessed on 23 July 2021. PR = proliferation, LB = low bone disease, MS = MMSET, HY = hyperdiploid, CD1 = cyclin D1 and cyclin D3, CD2 = cyclin D2 and cyclin D3, MF = MAF. Statistical difference was assayed using Student’s *t*-test. * *p* ≤ 0.05, ** *p* ≤ 0.01, *** *p* ≤ 0.005.

MM is the second most frequent hematological cancer [221]. It is characterized by accumulation of malignant PCs in the bone marrow. Like normal PCs, MM cells produce Ig at high levels. MM cells display genomic abnormalities, including recurrent translocations that involve the Ig heavy chain locus on chromosome 14q32 [222]. This results in fusion products composed of the IGH enhancer in front of an oncogene, such as MMSET, CCND3, CCND1, or MAF [18]. These translocations induce cyclin deregulation, leading to cell cycle activation [17]. Furthermore, deletion of chromosome 17p, including *TP53* gene deletion, is associated with poor MM outcome [18]. The deregulation of cell cycle resulting from these abnormalities induces replication stress that increases the overall rate of DNA damage in cancer cells [223]. Consequently, these cells must cope with high Ig synthesis while keeping up DNA replication. The transcriptional stress reported in MM colocalize with replicative stress resulting in genomic instability [9,224]. Accordingly, recurrent mutations have been identified in PC-specific highly transcribed genes including EGR1, XBP1, BTG2, DDX5 and NFKB1A [222,224]. Of interest, we identified that other genes frequently mutated in MM, such as CDKN2A, DNMT3A, FGFR1 and KMT2A, are highly transcribed in bone marrow plasma cells (Figure 2 and Figure S1) [225,226], reinforcing the idea that the high transcription level on these genes increases the probability of collisions with the replication machinery that would lead to DNA lesions and mutagenesis. Therefore, the ability to deal with TRCs may represent an actionable Achilles’ heel in MM. Comparison of the Affymetrix gene expression profiles of purified normal bone marrow PCs (*n* = 5) and purified MM cells from newly diagnosed patients (*n* = 206) [227] using the GenomicScape webtool [21] showed that 13 TRC resolution factors were significantly overexpressed in MM cell samples (fold change ≥2, false discovery rate <5%; 1000 permutations) (Figure 3). The CEL files and MAS5 files are available in the ArrayExpress public database (E-MTAB-372). Considering the supposed key role of TRC resolution in MM cells, we sought to identify TRC resolution factors associated with poor MM outcome. For this, we used the publicly available Affymetrix gene expression profiles (Gene Expression Omnibus, GSE2658) of a cohort of 345 purified MM cell samples of previously untreated patients from the University of Arkansas for Medical Sciences (Little Rock, AR, USA). Analysis with the MaxStat R function indicated that high expression of 9 of these 13 TRC resolution factors was associated with poor MM outcome (TT2 cohort, *n* = 345) (Figure 4). Strikingly, we found a significant overexpression of eight out of the nine TRC resolution genes in the high risk MM subgroup of patients Proliferation (PR subgroup) characterized by an overexpression of genes involved in cell cycle and proliferation (Figure 5) [228]. Altogether, this suggests that increasing TRCs could be a specific therapeutic strategy to kill malignant PCs. Possible strategies to do this are described in the following paragraphs.

## 4. TRC Targeting Approaches

First, G4 ligands have been developed to stabilize G4 structures. G4 stabilization induces DNA damage by increasing the collision rate between replication and transcription [229], or by promoting DNA repair-mediated DNA breaks [230]. Moreover, these ligands downregulate G4-containing oncogenes [231,232,233,234]. Indeed, G4 structures are found in the promoter of several oncogenes, such as c-MYC [235] and BCL2 [236,237]. Overall, these structures are rare in tumor-suppressor genes and frequent in proto-oncogenes [238]. A recent study showed that MYC-G4 stabilization using a small molecule G4 stabilizer in MM cells reduces MYC transcription and increases endoplasmic reticulum stress [239].

Pyridostatin is a very selective G4 ligand [240,241,242]. This molecule induces DNA damage, activates the DDR [45,240,241] and has been widely studied in different cancer types. Pyridostatin cytotoxicity could involve the trapping of Topoisomerase II on the chromatin [243]. In BRCA2-depleted cells, pyridostatin promotes telomere fragility and reduces proliferation by inducing DNA breaks. Of note, pyridostatin could overcome resistance to Olaparib in homologous recombination-defective cells [244]. Interestingly, a pyridostatin analog acts synergistically with DNA PK inhibitors, with greater effect in BRCA2-defective cells [245]. Moreover, pyridostatin reduce the transcriptional activity of the oncogenic EWSR1-FLI1 fusion product in Ewing sarcoma [246]. Pyridostatin can act by reducing the G4 unwinding activity of BLM and PIF1 [247,248], and also by altering splicing patterns [249]. Accordingly, pyridostatin impairs BRCA1 transcription in cultured neurons, possibly by stabilizing the G4 structures present in its gene promoter [250]. It also reduces the proliferation of neural stem cells and progenitor cells by inducing DNA damage and cell death [251].

Another G4 ligand, CX-5461, is currently being tested in an advanced phase I clinical trial for patients with BRCA-deficient tumors (NCT02719977). This molecule has p53-dependent anti-tumoral activity [252,253], impairs replication fork progression, and induces G4 accumulation in vivo. It could have a therapeutic effect in both NHEJ- and homologous recombination-deficient cancers [254]. It could be interesting to identify the patients who could benefit from a G4 ligand-based therapy. Indeed, it has been reported that high expression of the TREX1 component THOC2 is associated with higher resistance to CX-5461 [255]. Conversely, FANCJ mutations can sensitize cells to this compound [128].

Second, R-loops could be targeted [255] with small molecule intercalators, such as ethidium bromide or actinomycin D [256,257], and with G4 ligands that can modulate their stability. It is also possible to target RNA:DNA hybrid binding proteins [258,259,260]. Another strategy could be to target the spliceosome since it was shown that the modification of splicing patterns can promote R-loop accumulation or dysregulation [229,261,262,263]. Additionally, the anti-tumor drug trabectedin inhibits cell proliferation and induces genome instability in an R-loop-dependent manner [264].

Finally, inhibitors of TRC resolution factors could be of therapeutic interest in MM (Figure 2B). The finding that many of these factors are upregulated in MM cells suggests that these cells must cope with high TRC amounts. RNase H2 inhibitors have been developed [265], and a study showed that this enzyme could constitute a new anti-cancer drug target [266]. Several DNA helicase inhibitors also are available, for example for BLM [267] and DNA2 [268]. Some anti-cancer drugs, such as DNA intercalating agents and DNA minor groove binding drugs, inhibit BLM activity [269]. In glioma cells, Ivermectin inhibits DDX23 helicase activity and suppresses cell proliferation in vivo [270]. Mirin is a pharmacological inhibitor of MRE11 [271,272]. The anti-obesity drug celastrol induces FANCD2 degradation by the proteasome, and shows anti-cancer activity in vitro and in vivo [273], and other FA pathway inhibitors are commercially available [274,275]. The PRMT5 inhibitor EPZ015666 demonstrated anti-MM activity in both cell lines and primary cells from patients, without affecting normal PBMCs viability [109]. The common flavonoid luteolin reduces hepatocellular carcinoma cell proliferation in mice and increases cisplatin sensitivity through THOC1 inhibition [82,276]. HAMNO is a small-molecule inhibitor of RPA that induces replication stress specifically in cancer cells and acts in synergy with etoposide [277]. Further investigations are mandatory to demonstrate the therapeutic interest of TRC resolution genes inhibitors. This may be instrumental for the development of novel tools and strategies in the treatment of MM patients taking advantage of R-loops and G4s to selectively increase replication stress in MM cells while avoiding selecting variants’ subclones.

## 5. Conclusions

Despite therapeutic advances and the increase in overall survival, there is still no definitive treatment for MM, and most patients invariably relapse after several lines of treatment. Therefore, new strategies to overcome treatment resistance are urgently needed. A series of complex molecular events are involved in MM development, such as chromosomal and genomic abnormalities leading to oncogene activation and deregulation of signaling pathways involved in cell survival and proliferation (NF-kB and RAS signaling). However, the exact pathophysiological mechanism leading to myelomagenesis (i.e., the transformation of a normal PC in a malignant PC) remains unclear. Thus, identifying the precise stage of PC differentiation involved in MM initiation would bring critical knowledge on this cancer, leading to potential innovative therapeutic strategies. Furthermore, MM is characterized by a strong molecular and clinical heterogeneity. The development of targeted therapies and precision medicine that take into account the tumor clonality of each patient should be a promising strategy.

Antibody secretion, the key biological function of PCs, starts at the prePB stage of B-cell differentiation and is maintained in malignant PCs. This underlines the fact that the transcription rate in both prePBs and malignant PCs is elevated. PrePBs exhibit the highest proliferation rate, and malignant PCs face replication stress induced by overexpression of oncogenes that deregulate the cell cycle. Consequently, transcription and replication in prePBs and in malignant PCs need to be especially tightly coordinated to avoid too many interferences that would increase replication stress and genomic instability over a threshold compatible with cell survival. A failure to cope with these interferences could have a significant impact on replication stress and mutagenesis involved in myelomagenesis. Furthermore, participation in successive GC reactions could result in cumulative acquisition of further off-target mutations in these cells [219,220]. On the other hand, these effects might open up the therapeutic possibility of TRCs enhancement to specifically kill malignant PCs.

## Supplementary Materials

The following are available online at https://www.mdpi.com/article/10.3390/cancers13153755/s1, Figure S1: 33 genes frequently mutated in MM are overexpressed in bone marrow plasma cells (BMPCs), Table S1: 83 TRCs resolution genes identified in the literature, Table S2: 41 TRCs resolution genes significantly overexpressed in pre-plasmablasts compared to memory B cells, plasmablasts and plasma cells.
