# Supplementary material for: Transcription/Replication Conflicts in Tumorigenesis and Their Potential Role as Novel Therapeutic Targets in Multiple Myeloma
Source: Cancers (Basel). 2021 Jul 27;13(15):3755. doi: 10.3390/cancers13153755 (PMC8345052; doi:10.3390/cancers13153755)
Supplement: Supplementary file 1 [file cancers-13-03755-s001.zip › cancers-1238831-Supplementary.pdf]

cancers
MDPI

Supplementary Materials: Transcription/Replication Conflicts in Tumorigenesis and Their Potential Role as Novel Therapeutic Targets in Multiple Myeloma

Laure Dutrieux, Yea-Lih Lin, Malik Lutzmann, Raphaël Rodriguez, Michel Cogné, Philippe Pasero and Jérôme Moreaux

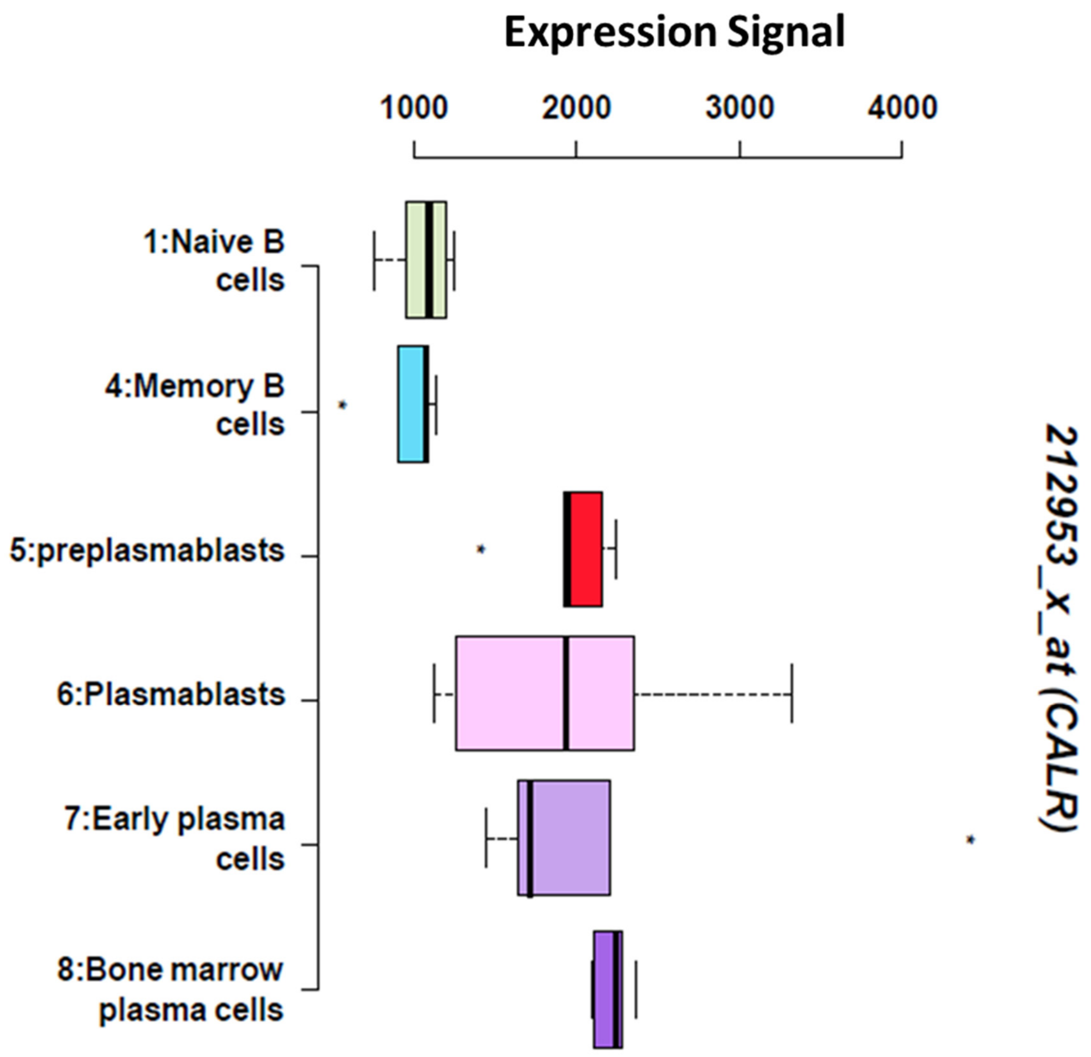

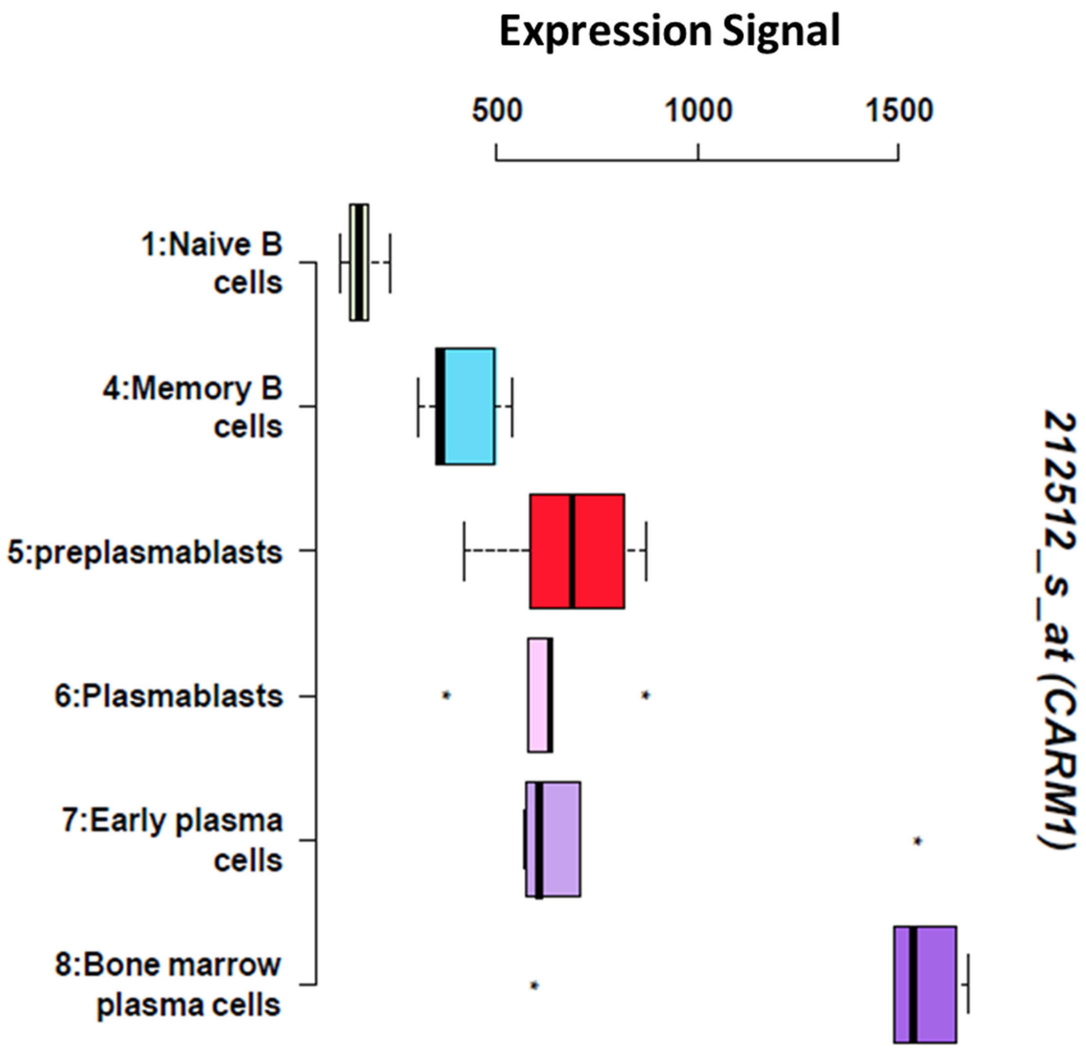
Expression Signal
500
1000
1500
1:Naive B cells
4:Memory B cells
5:preplasmablasts
6:Plasmablasts
7:Early plasma cells
8:Bone marrow plasma cells
212512_s_at (CARM1)

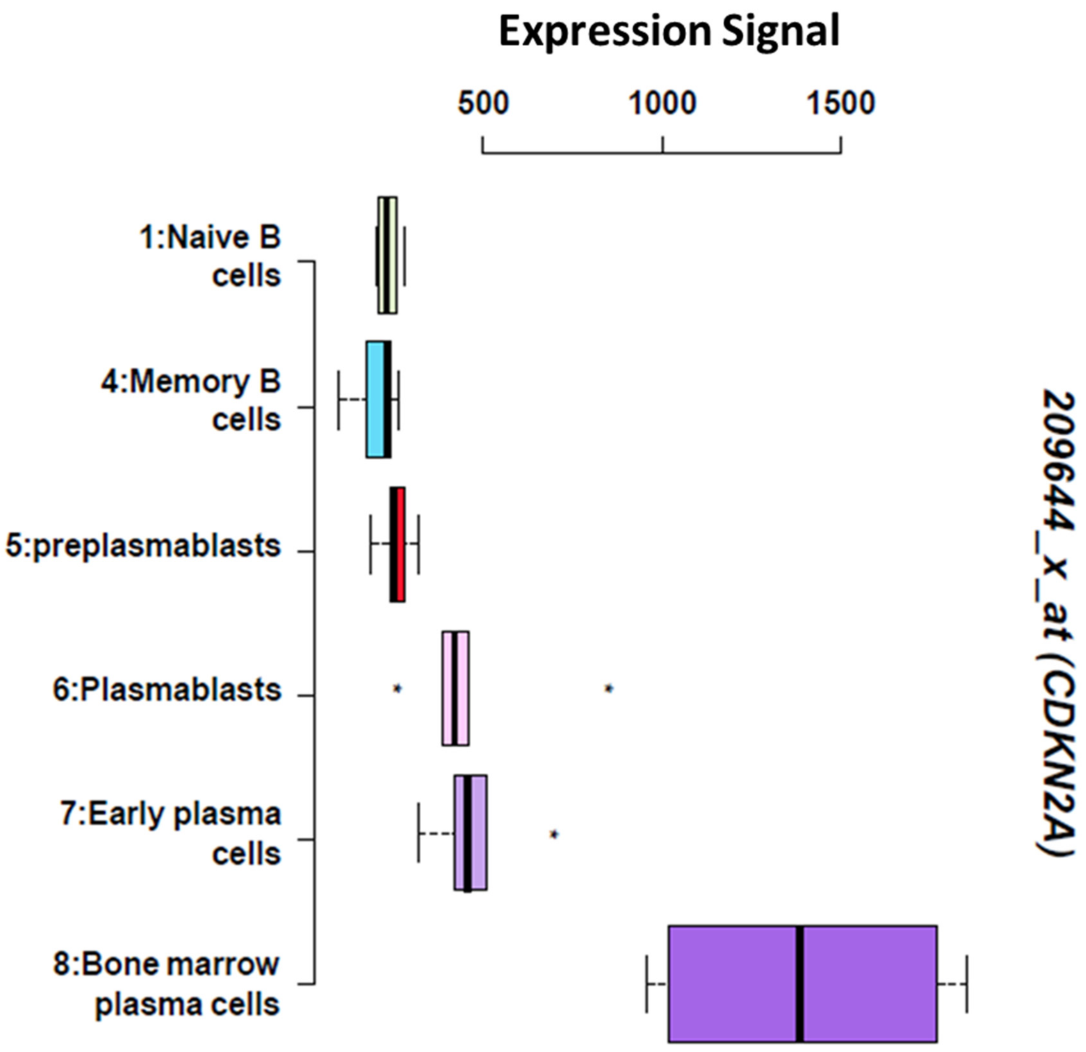

Expression Signal
500
1000
1500
1:Naive B cells
4:Memory B cells
5:preplasmablasts
6:Plasmablasts
7:Early plasma cells
8:Bone marrow plasma cells
209644_x_at (CDKN2A)

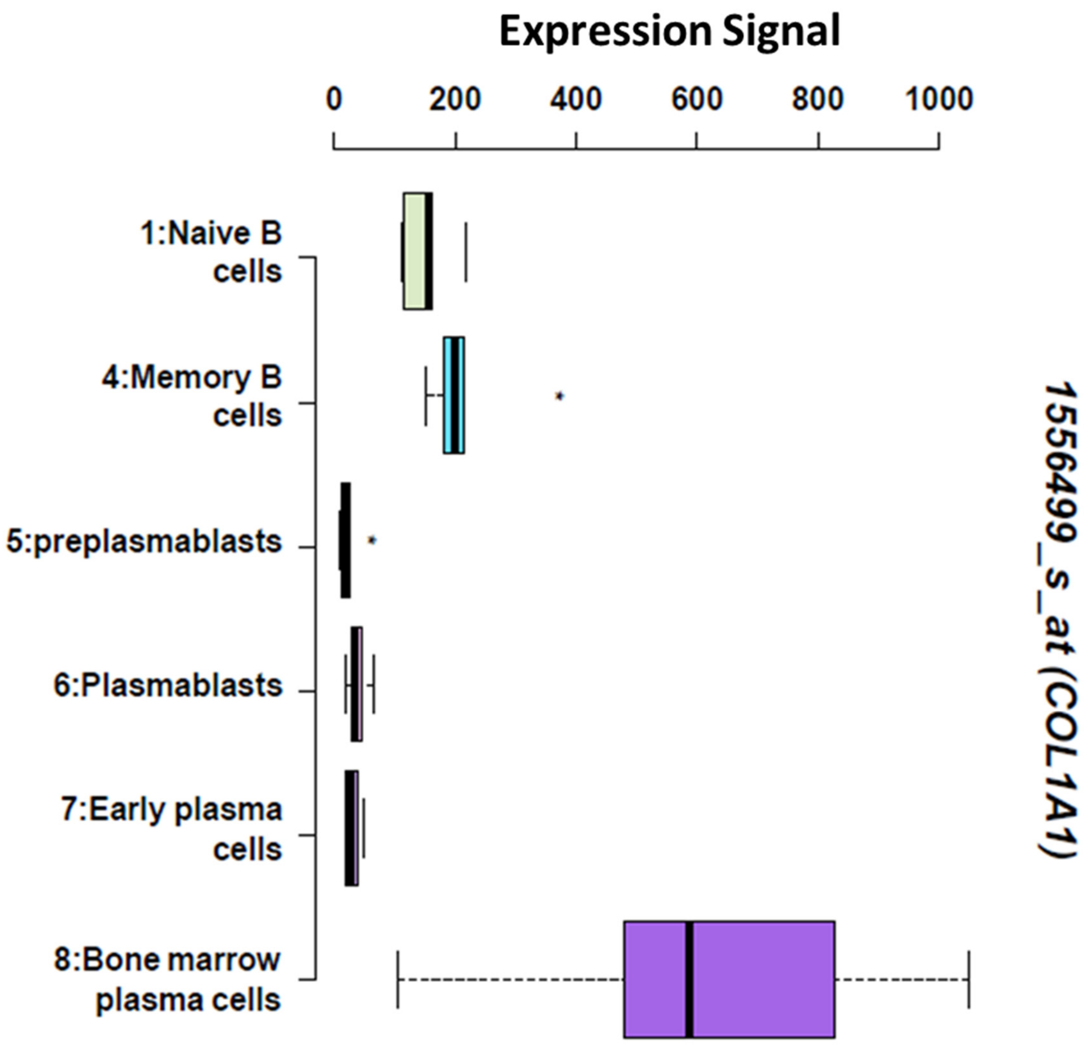

Expression Signal
0
200
400
600
800
1000
1:Naive B cells
4:Memory B cells
5:preplasmablasts
6:Plasmablasts
7:Early plasma cells
8:Bone marrow plasma cells
1556499_s_at (COL1A1)

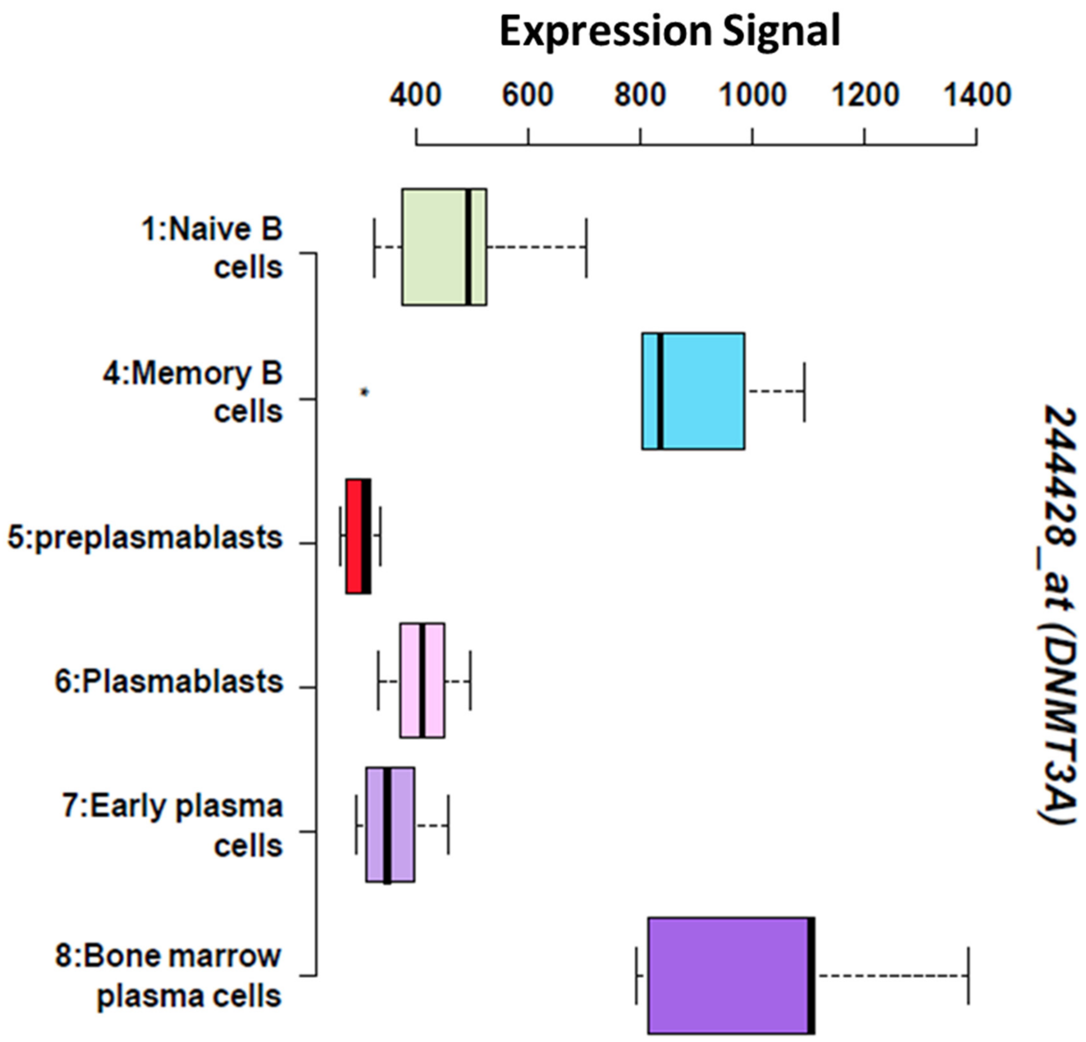
Expression Signal
400
600
800
1000
1200
1400
1:Naive B cells
4:Memory B cells
5:preplasmablasts
6:Plasmablasts
7:Early plasma cells
8:Bone marrow plasma cells
244428_at (DNMT3A)

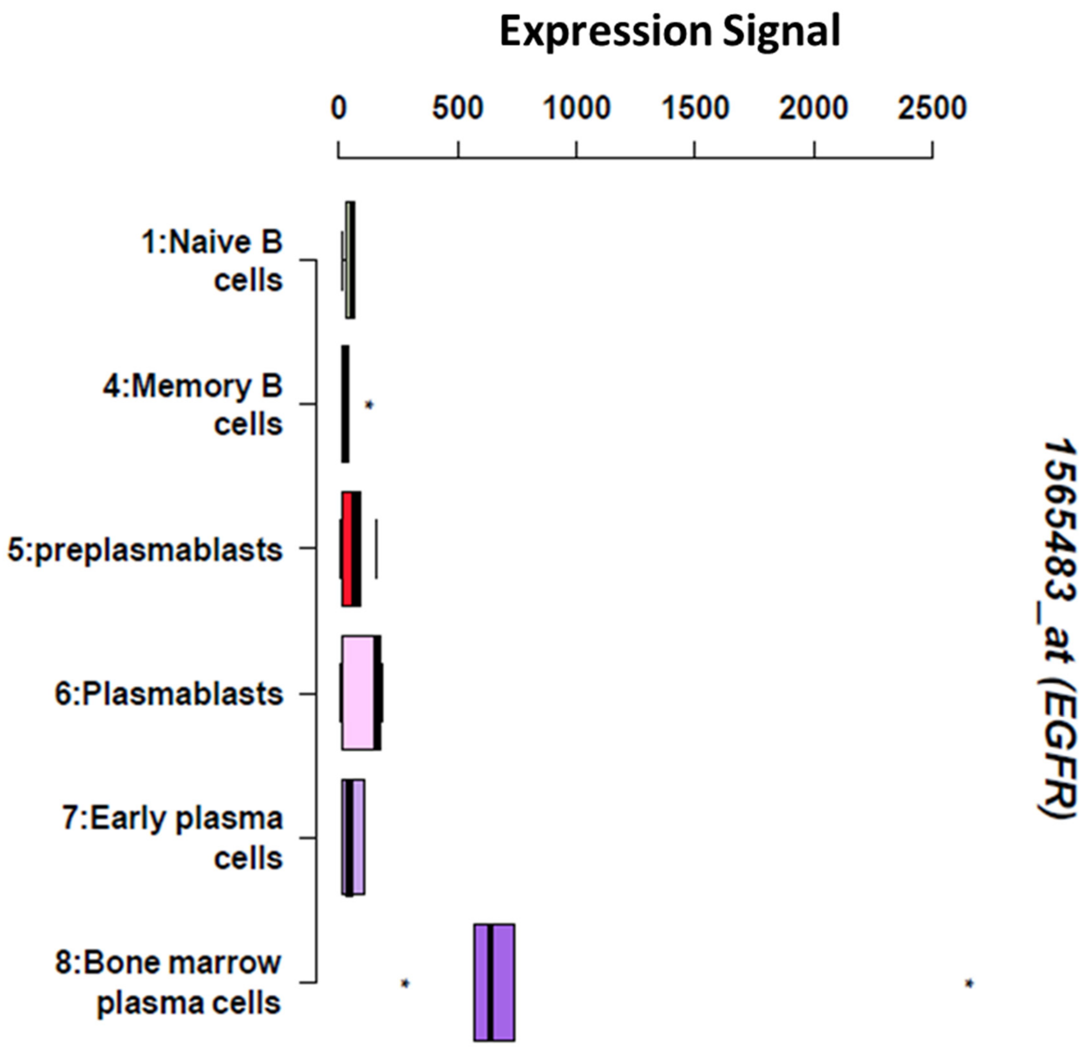
Expression Signal
0
500
1000
1500
2000
2500
1:Naive B cells
4:Memory B cells
5:preplasmablasts
6:Plasmablasts
7:Early plasma cells
8:Bone marrow plasma cells
1565483_at (EGFR)

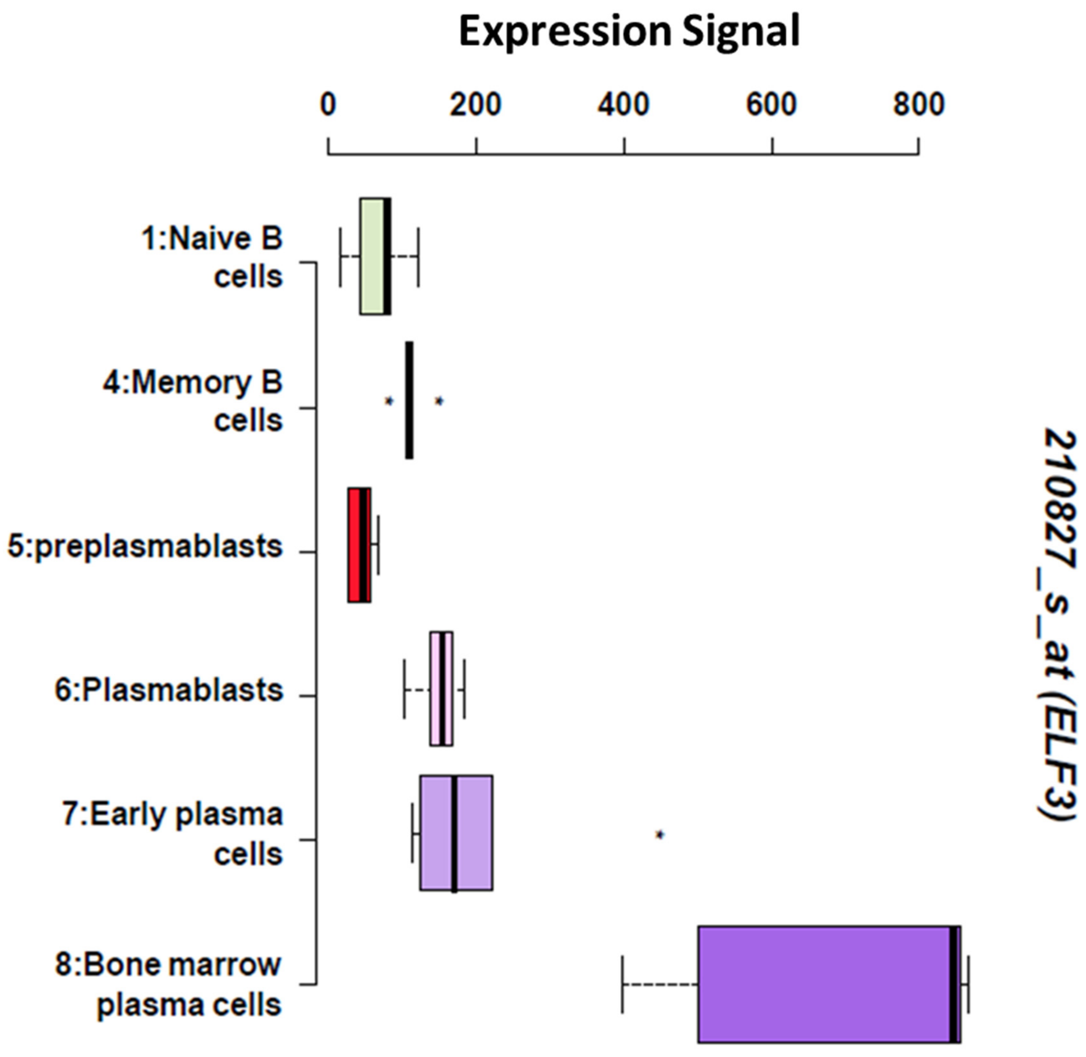
Expression Signal
0
200
400
600
800
1:Naive B cells
4:Memory B cells
5:preplasmablasts
6:Plasmablasts
7:Early plasma cells
8:Bone marrow plasma cells
210827_s_at (ELF3)

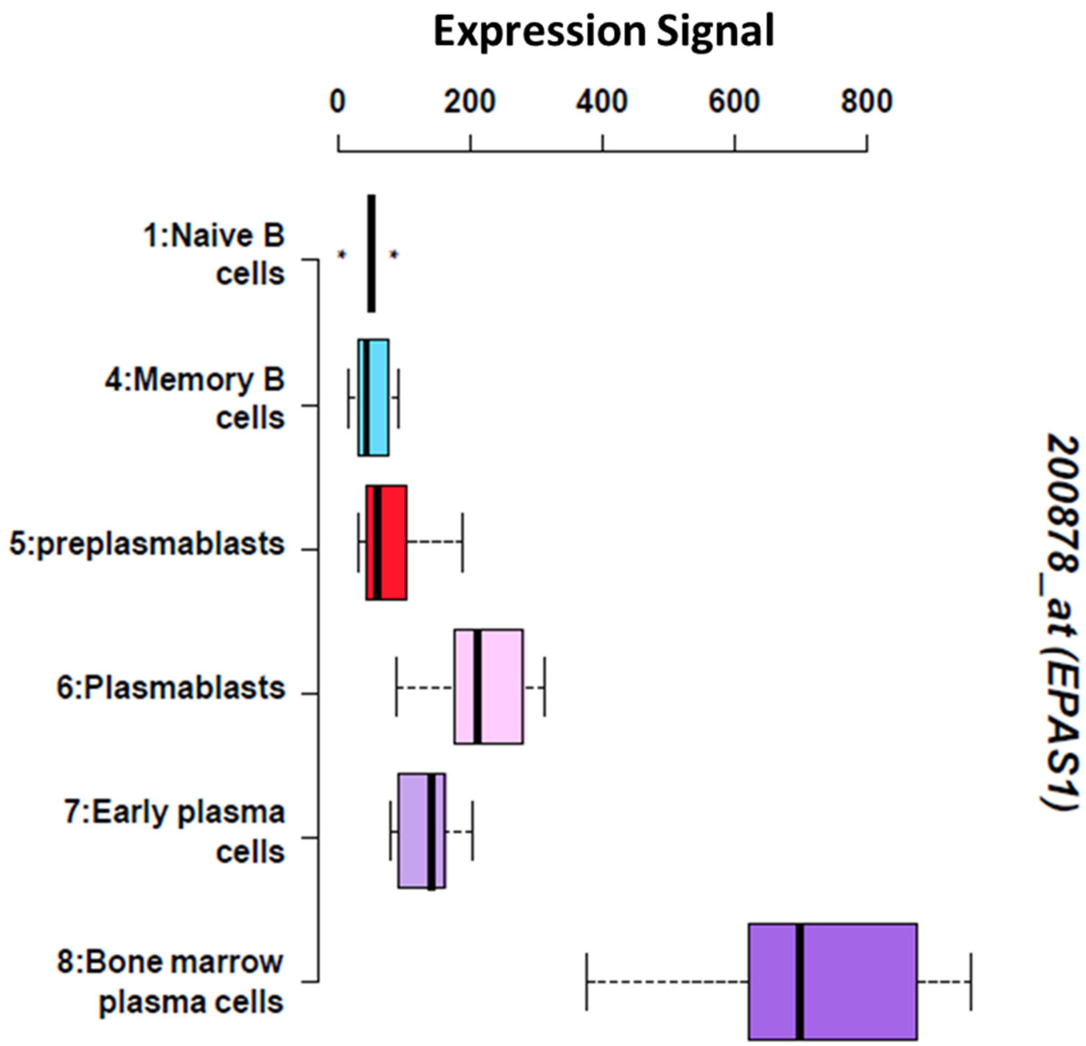
Expression Signal
0
200
400
600
800
1:Naive B cells
4:Memory B cells
5:preplasmablasts
6:Plasmablasts
7:Early plasma cells
8:Bone marrow plasma cells
200878_at (EPAS1)

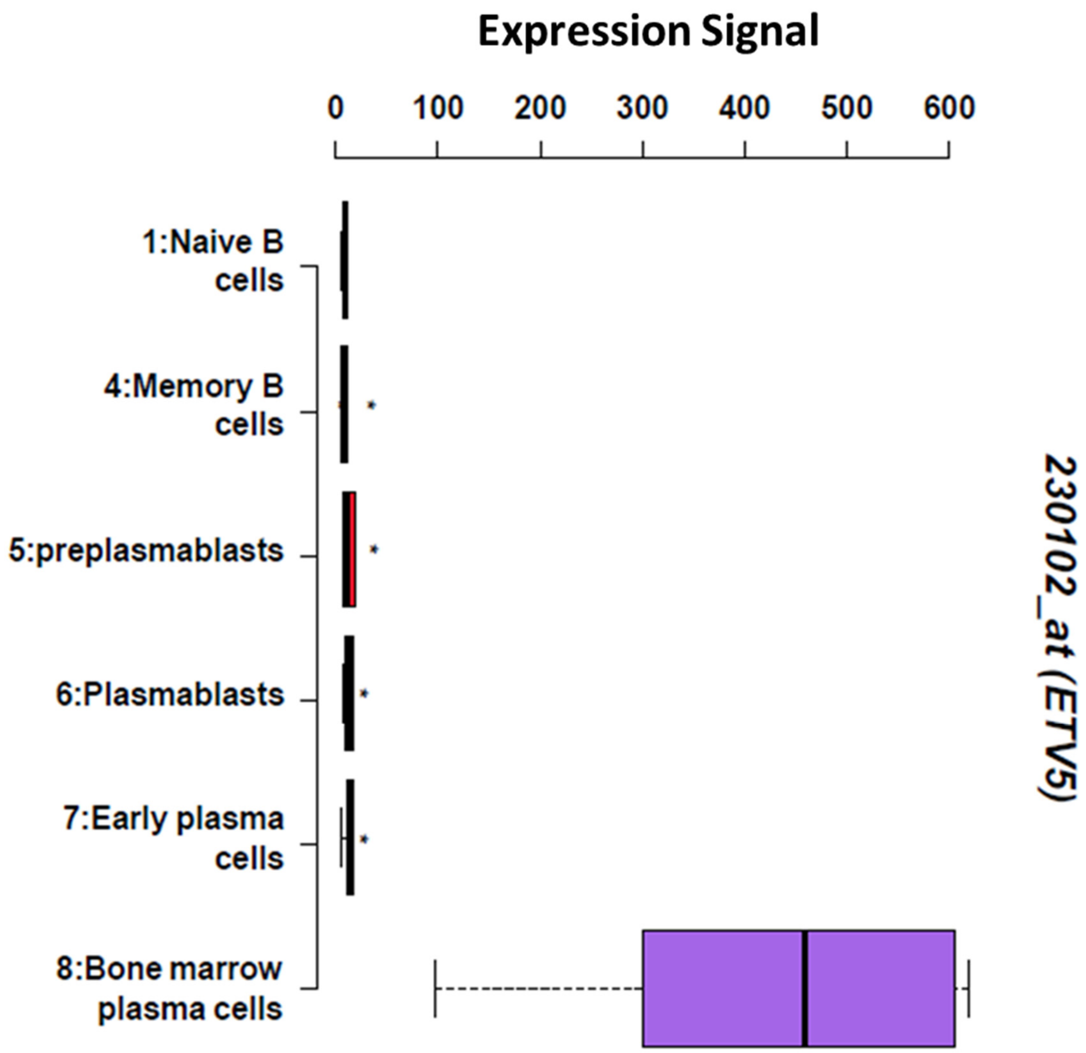
Expression Signal
0
100
200
300
400
500
600
1:Naive B cells
4:Memory B cells
5:preplasmablasts
6:Plasmablasts
7:Early plasma cells
8:Bone marrow plasma cells
230102_at (ETV5)

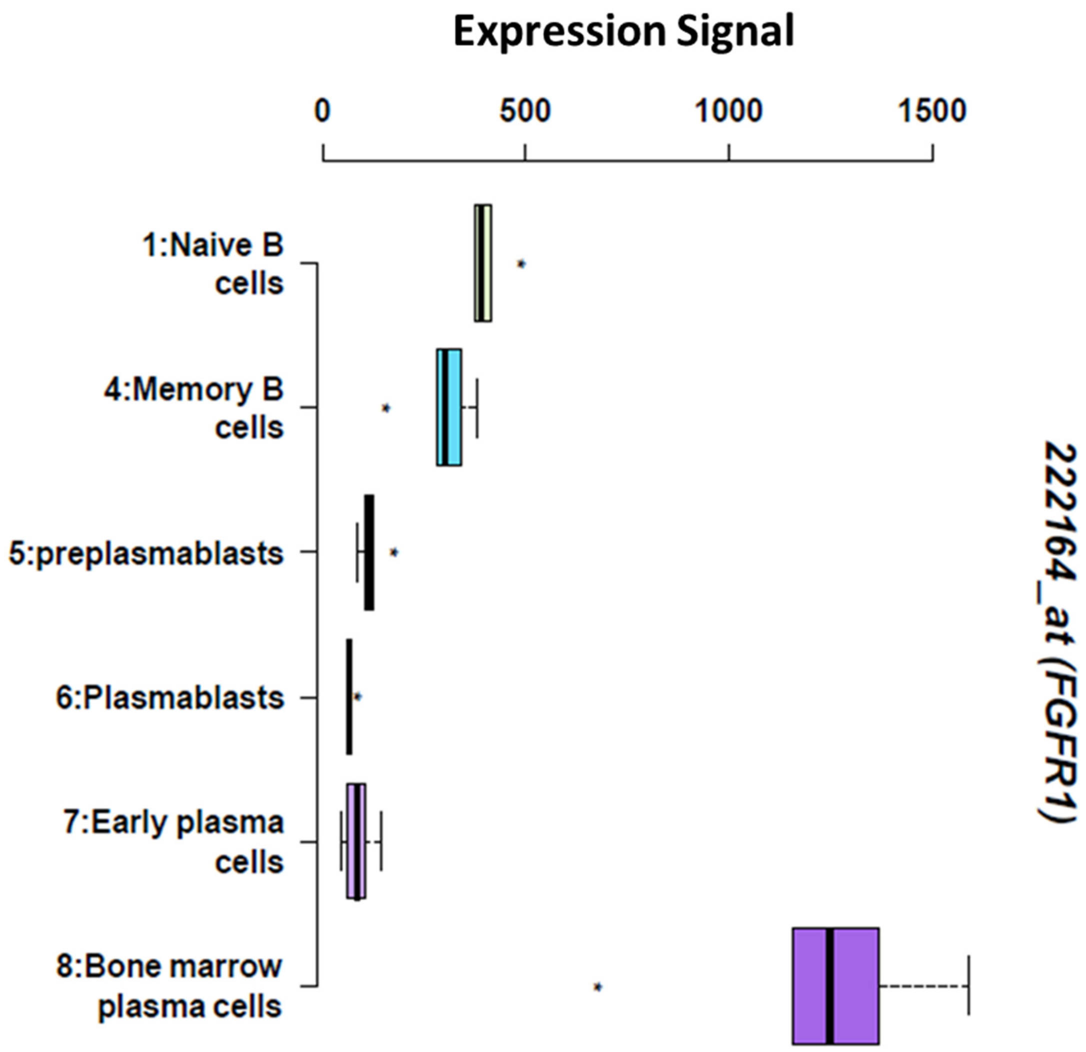
Expression Signal
0
500
1000
1500
1:Naive B cells
4:Memory B cells
5:preplasmablasts
6:Plasmablasts
7:Early plasma cells
8:Bone marrow plasma cells
222164_at (FGFR1)

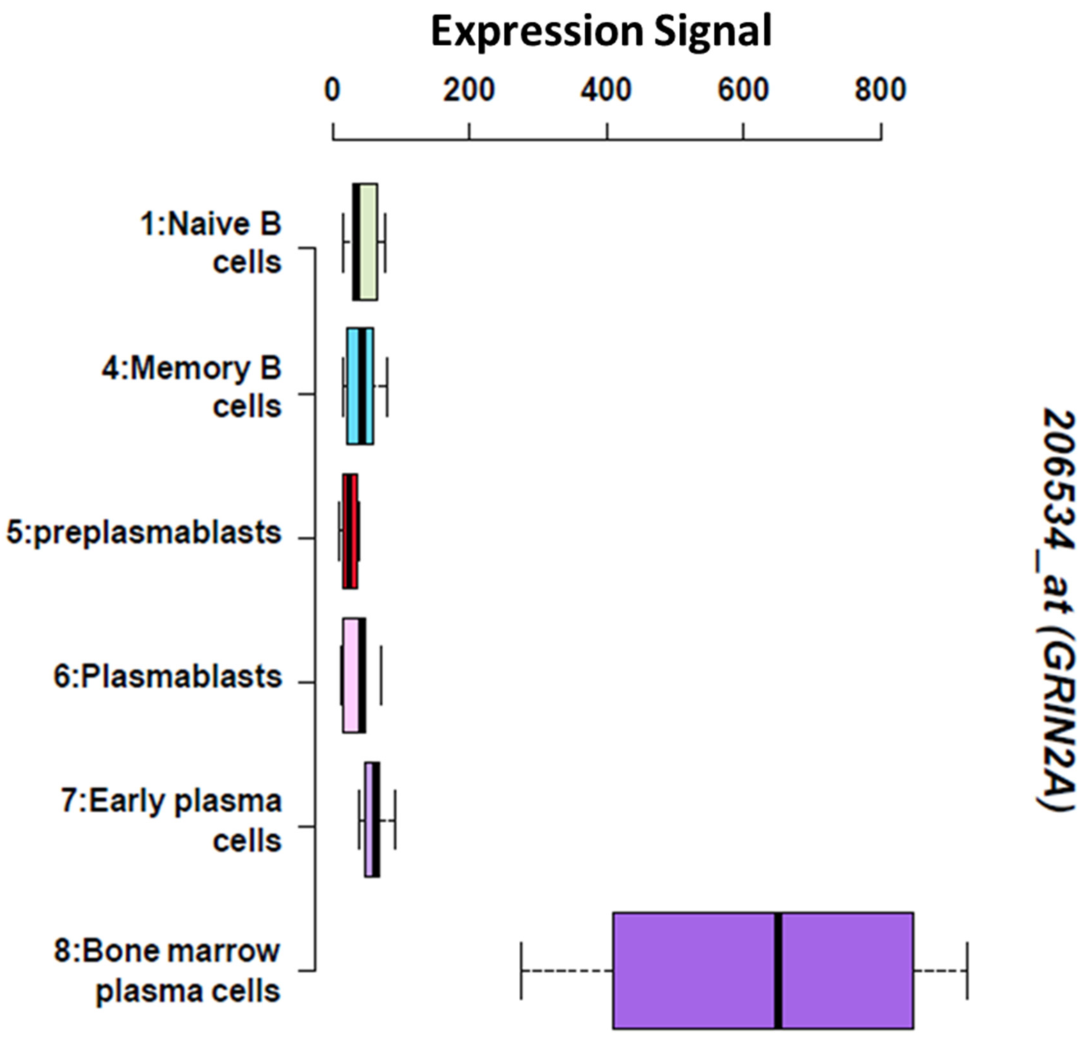
Expression Signal
0
200
400
600
800
1:Naive B cells
4:Memory B cells
5:preplasmablasts
6:Plasmablasts
7:Early plasma cells
8:Bone marrow plasma cells
206534_at (GRIN2A)

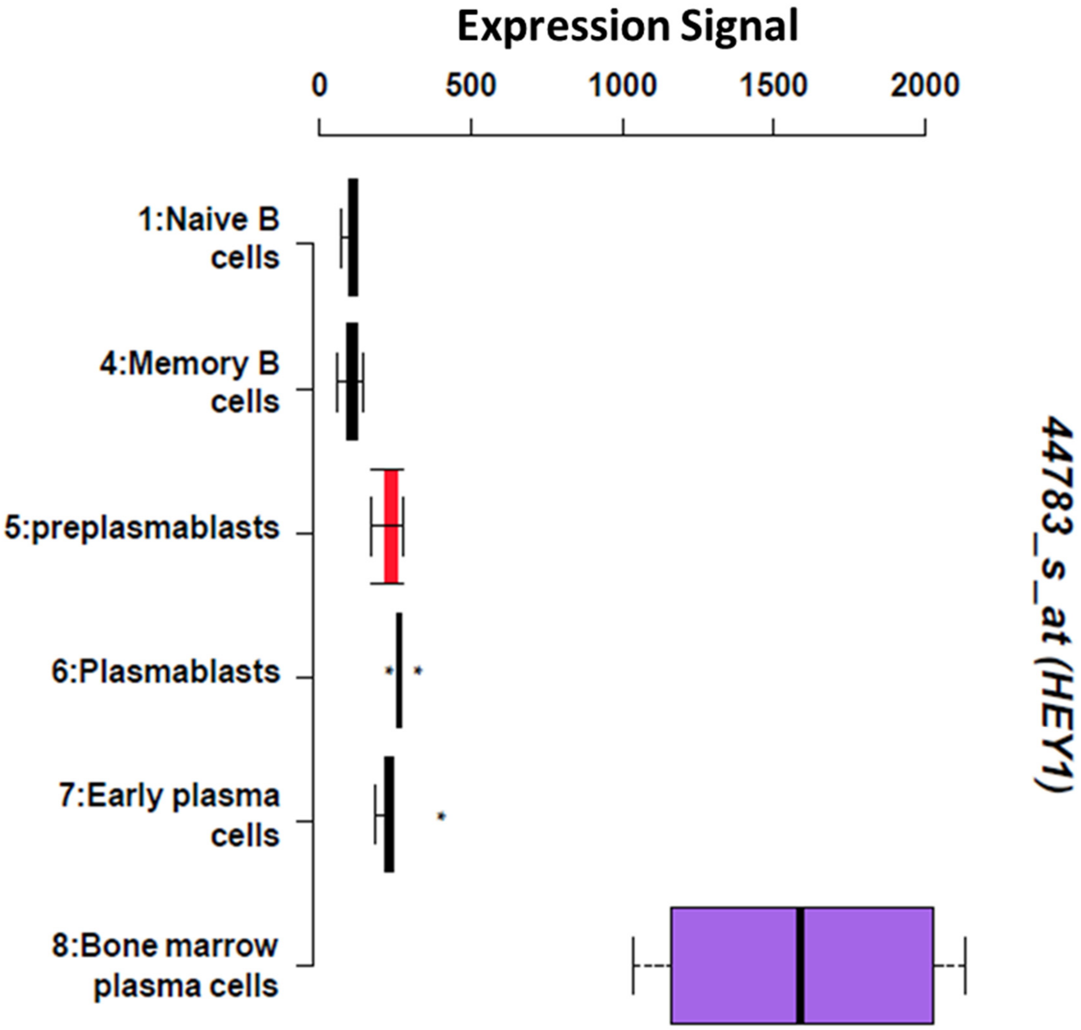
Expression Signal
0
500
1000
1500
2000
1:Naive B cells
4:Memory B cells
5:preplasmablasts
6:Plasmablasts
7:Early plasma cells
8:Bone marrow plasma cells
44783_s_at (HEY1)

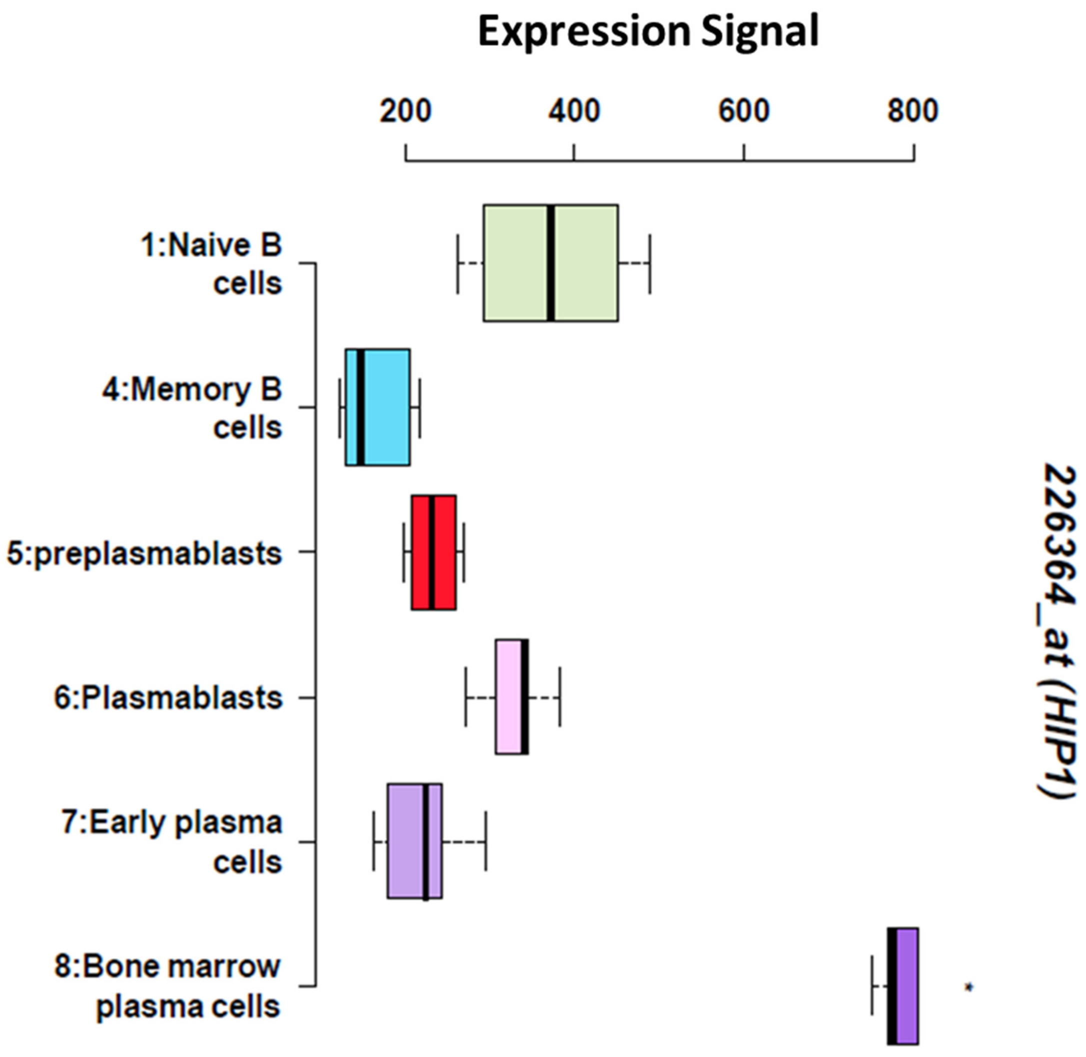
Expression Signal
200
400
600
800
1:Naive B cells
4:Memory B cells
5:preplasmablasts
6:Plasmablasts
7:Early plasma cells
8:Bone marrow plasma cells
226364_at (HIP1)

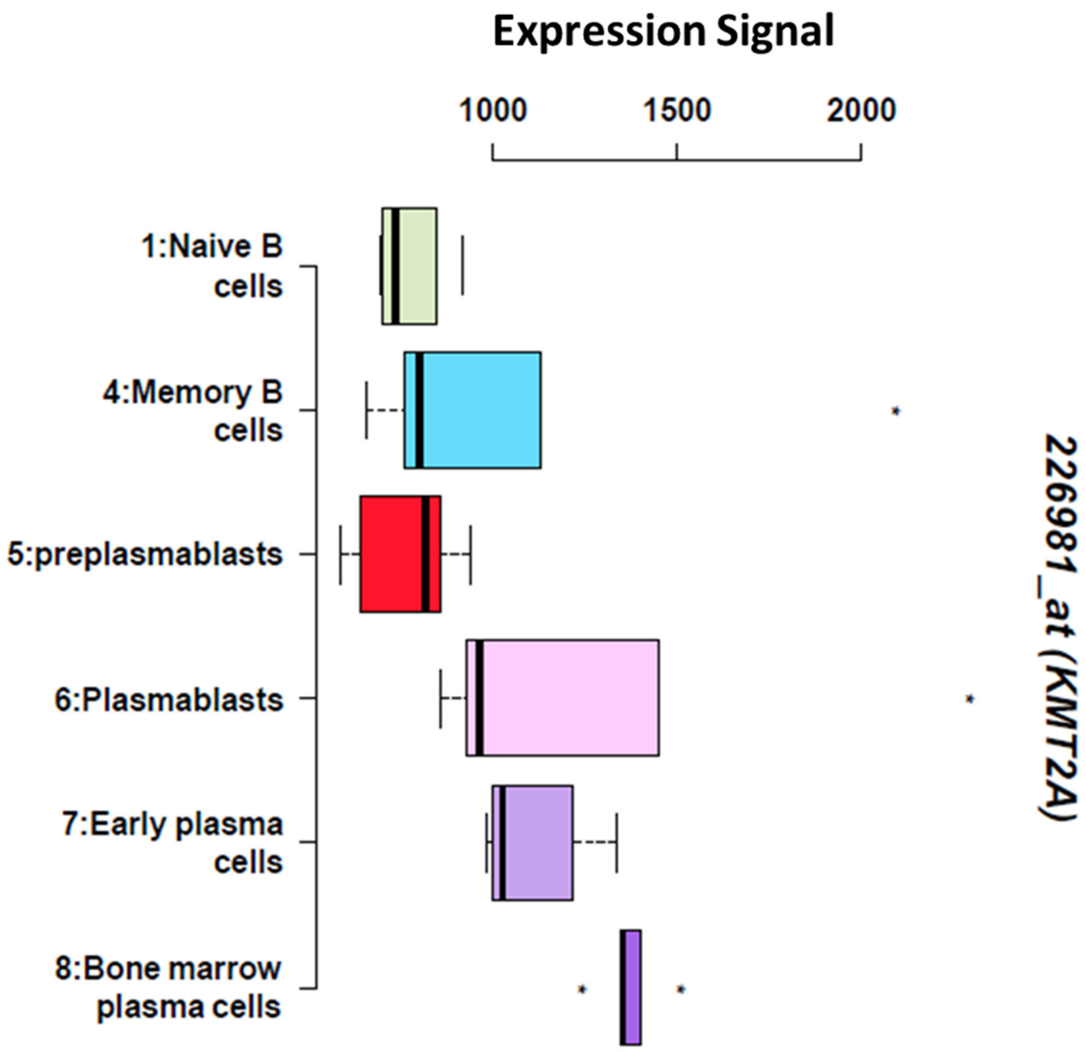

Expression Signal
1000
1500
2000
1:Naive B cells
4:Memory B cells
5:preplasmablasts
6:Plasmablasts
7:Early plasma cells
8:Bone marrow plasma cells
226981_at (KMT2A)

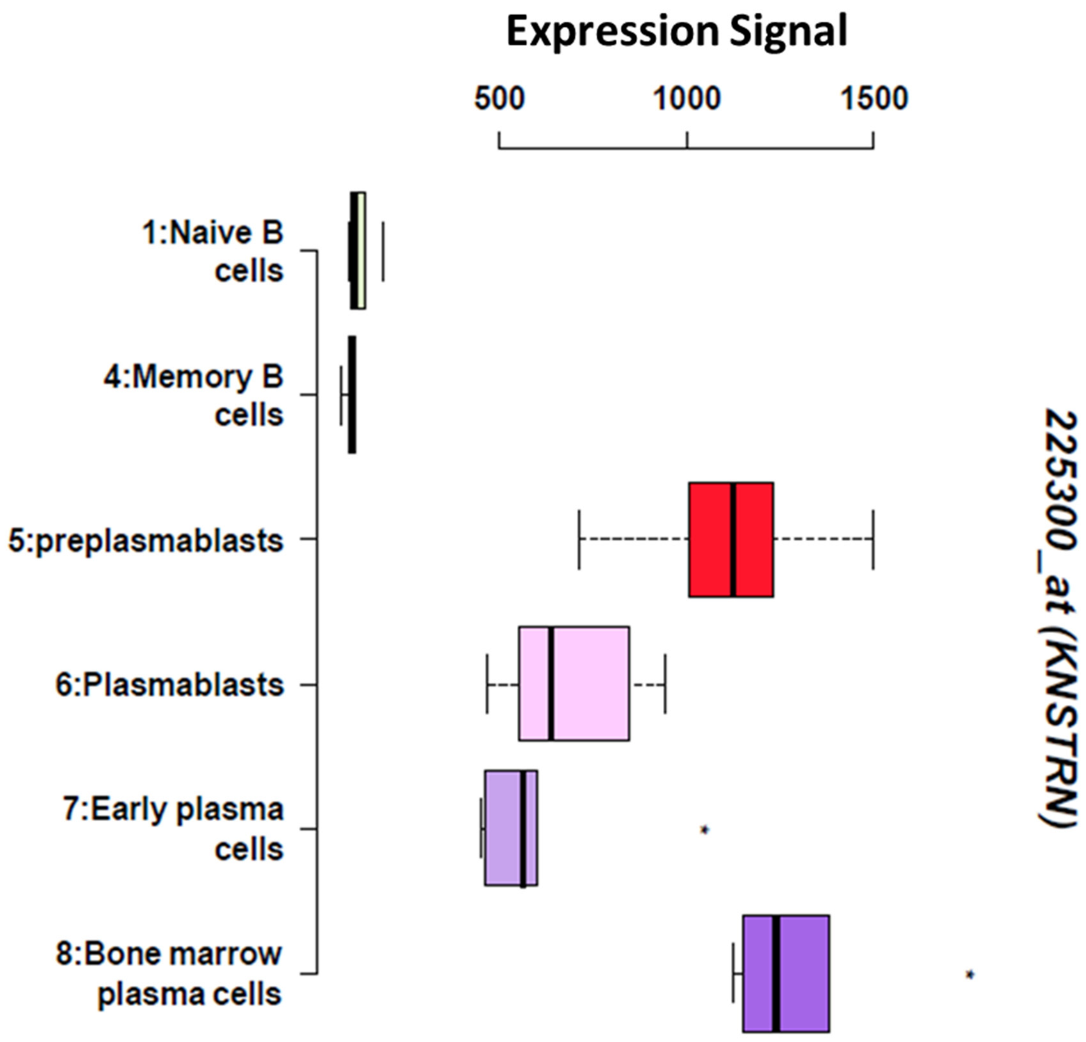

Expression Signal
500
1000
1500
1:Naive B cells
4:Memory B cells
5:preplasmablasts
6:Plasmablasts
7:Early plasma cells
8:Bone marrow plasma cells
225300_at (KNSTRN)

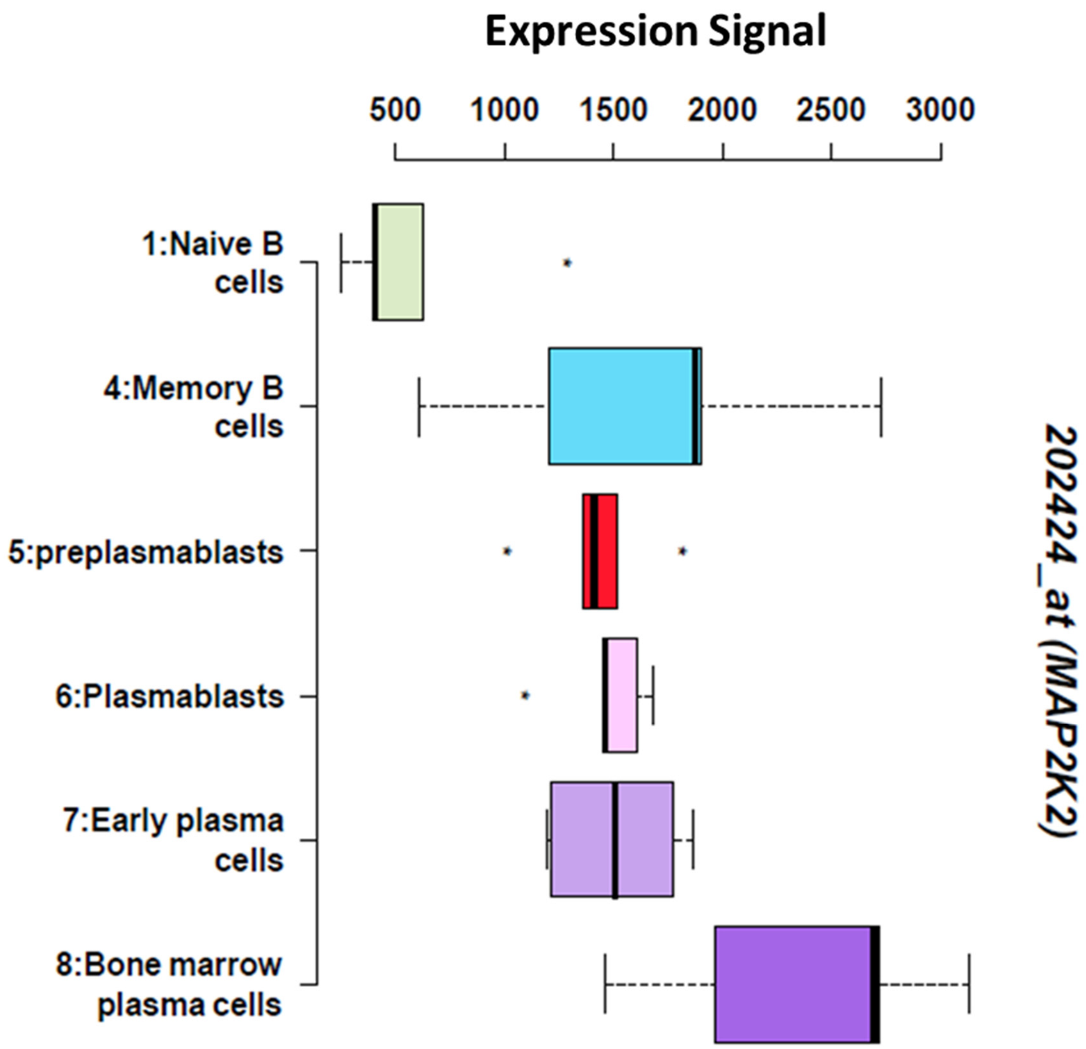
Expression Signal
500
1000
1500
2000
2500
3000
1:Naive B cells
4:Memory B cells
5:preplasmablasts
6:Plasmablasts
7:Early plasma cells
8:Bone marrow plasma cells
202424_at (MAP2K2)

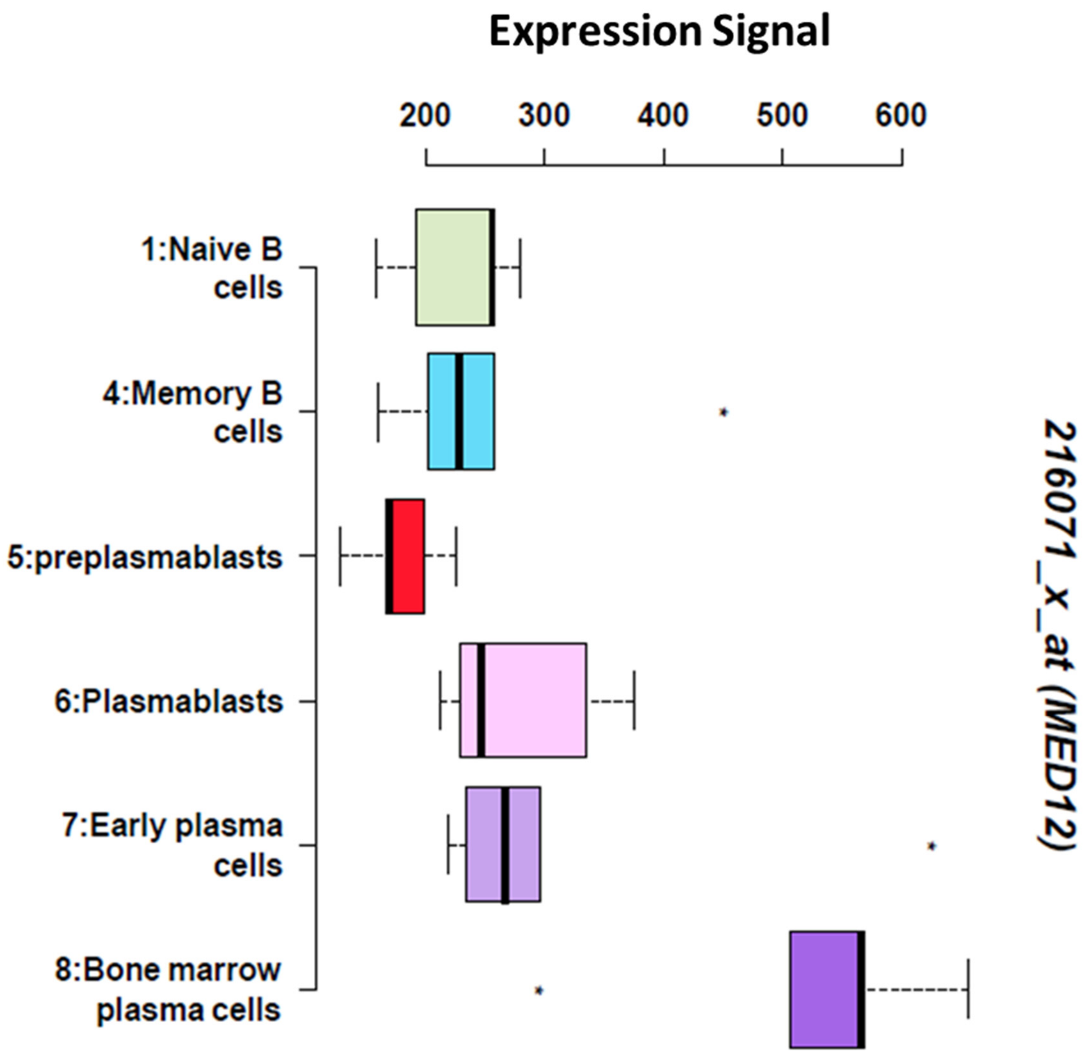

Expression Signal
200
300
400
500
600
1:Naive B cells
4:Memory B cells
5:preplasmablasts
6:Plasmablasts
7:Early plasma cells
8:Bone marrow plasma cells
216071_x_at (MED12)

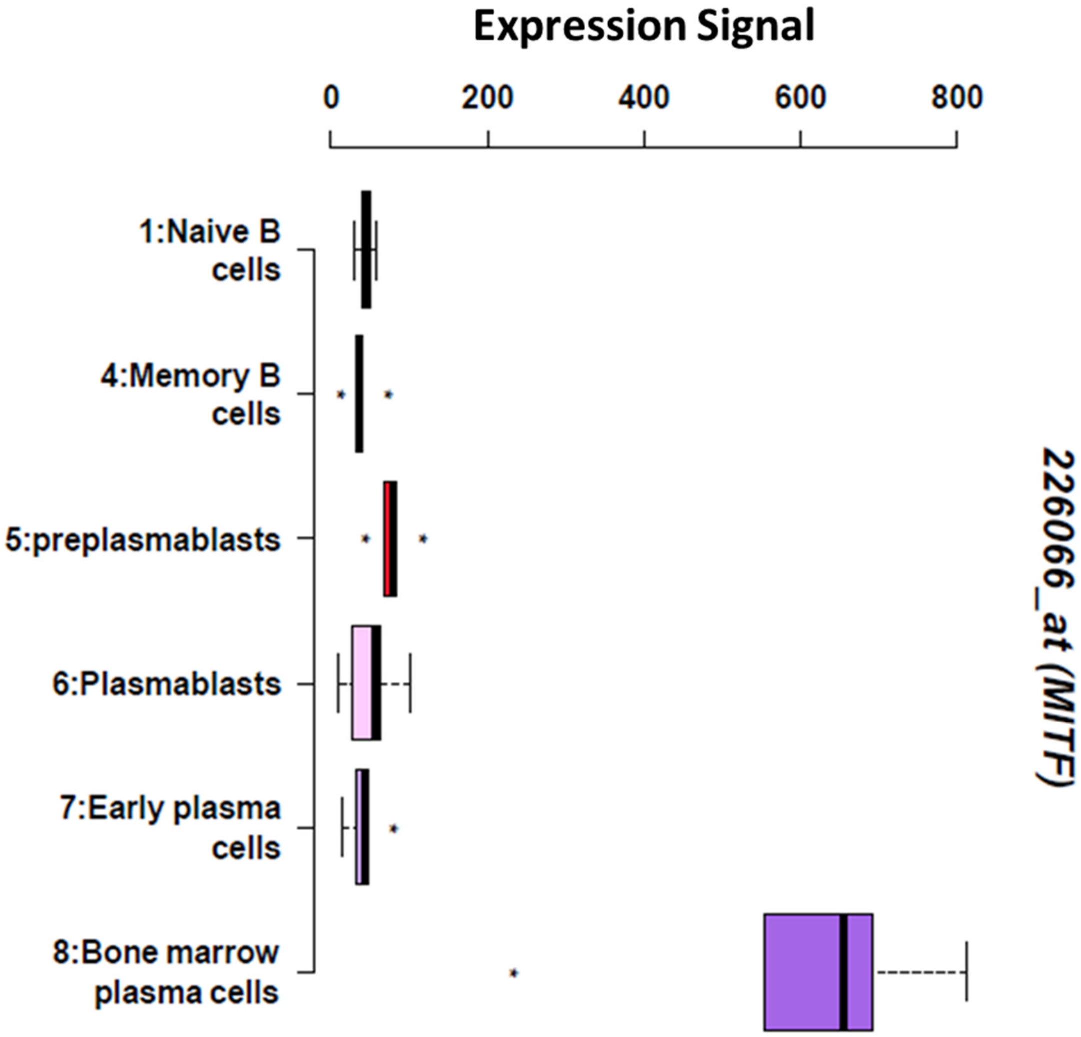
Expression Signal
0
200
400
600
800
1:Naive B cells
4:Memory B cells
5:preplasmablasts
6:Plasmablasts
7:Early plasma cells
8:Bone marrow plasma cells
226066_at (MITF)

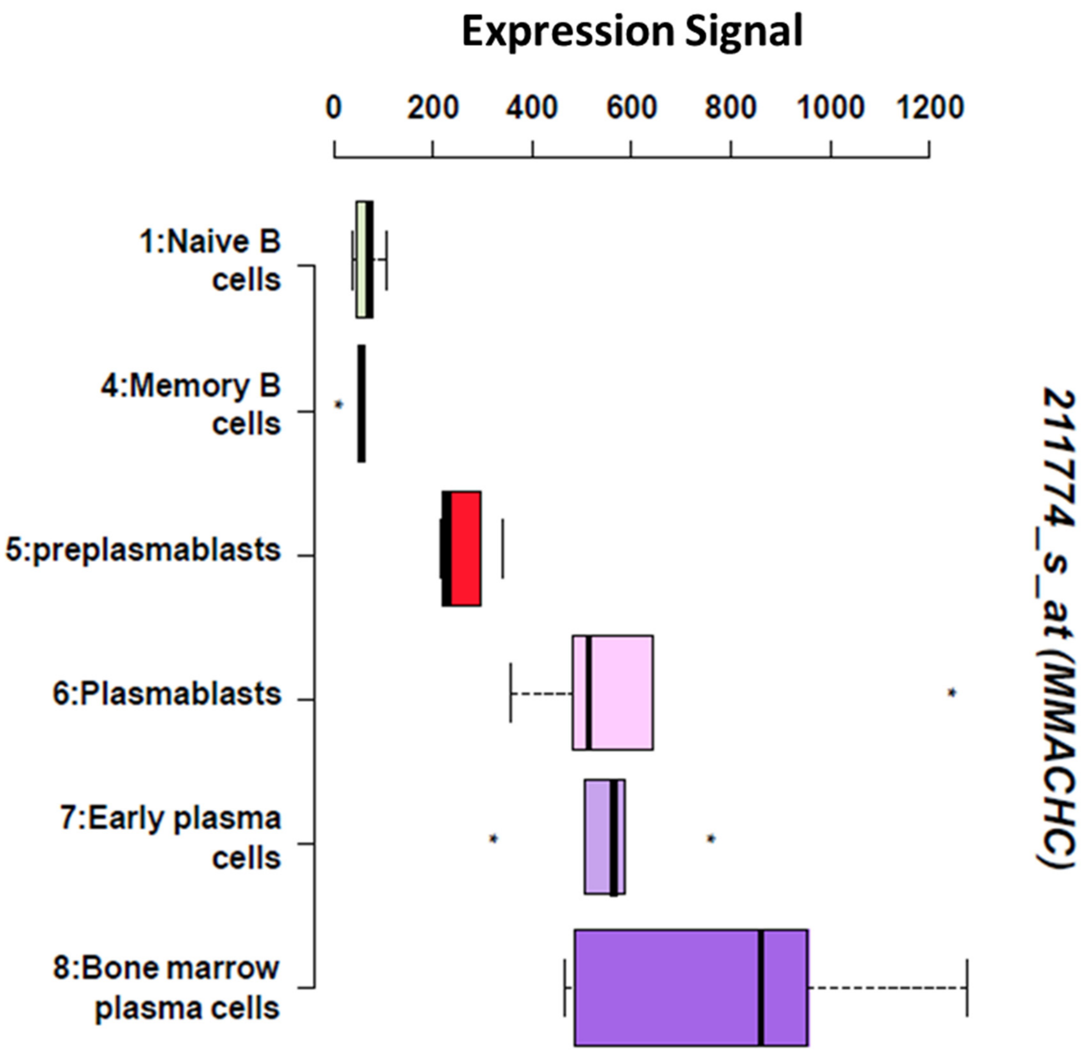
Expression Signal
0
200
400
600
800
1000
1200
1:Naive B cells
4:Memory B cells
5:preplasmablasts
6:Plasmablasts
7:Early plasma cells
8:Bone marrow plasma cells
211774_s_at (MMACHC)

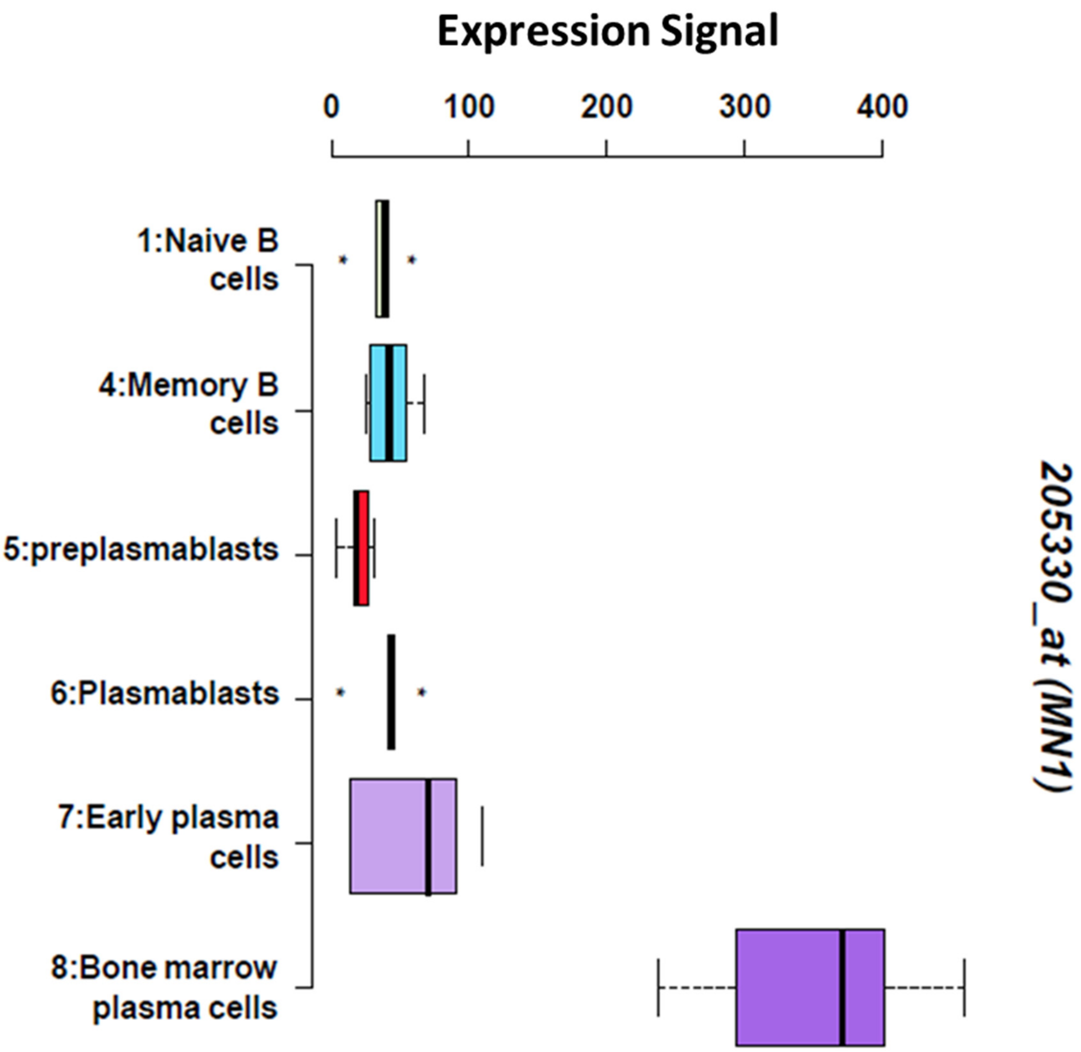

Expression Signal
0
100
200
300
400
1:Naive B cells
4:Memory B cells
5:preplasmablasts
6:Plasmablasts
7:Early plasma cells
8:Bone marrow plasma cells
205330_at (MN1)

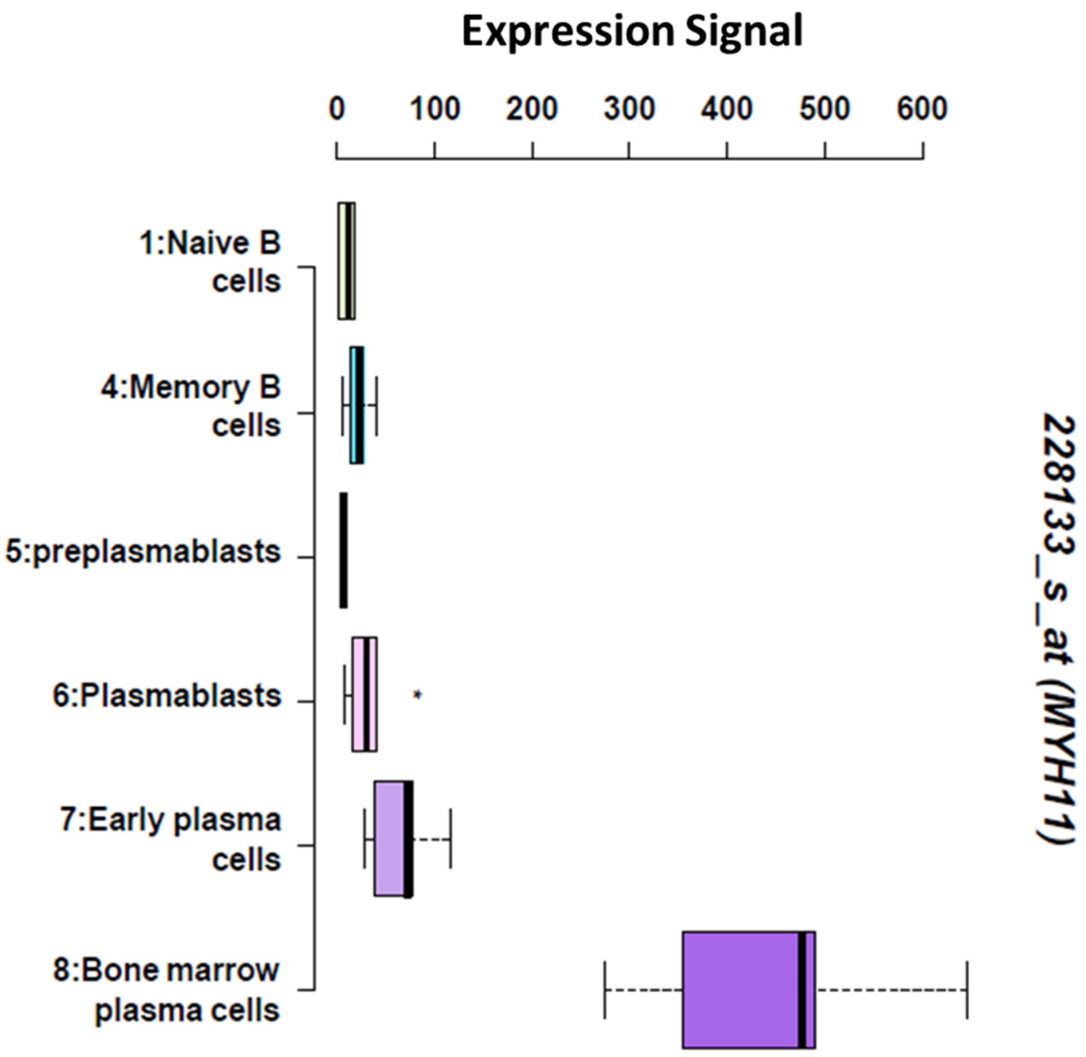

Expression Signal
0
100
200
300
400
500
600
1:Naive B cells
4:Memory B cells
5:preplasmablasts
6:Plasmablasts
7:Early plasma cells
8:Bone marrow plasma cells
228133_s_at (MYH11)

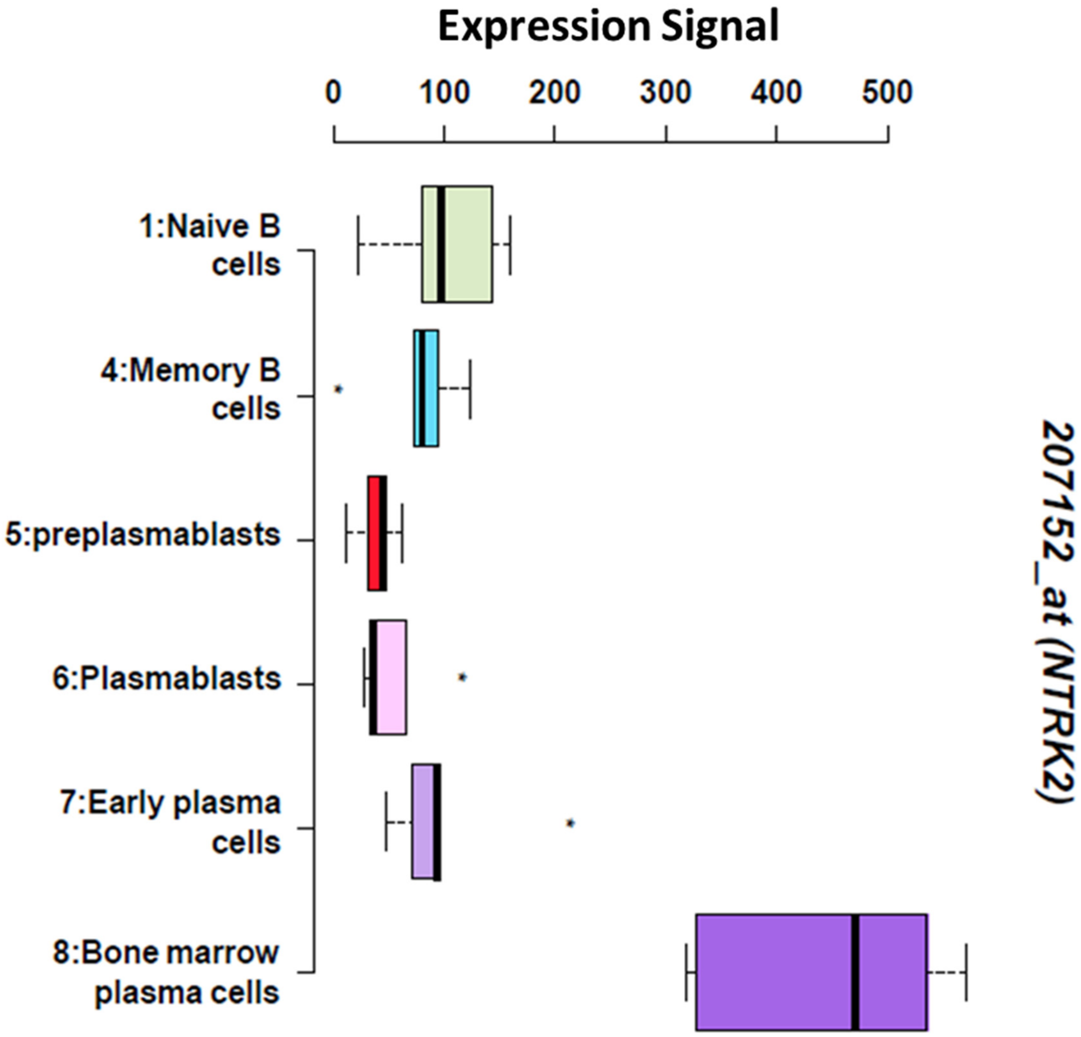

Expression Signal
0
100
200
300
400
500
1:Naive B cells
4:Memory B cells
5:preplasmablasts
6:Plasmablasts
7:Early plasma cells
8:Bone marrow plasma cells
207152_at (NTRK2)

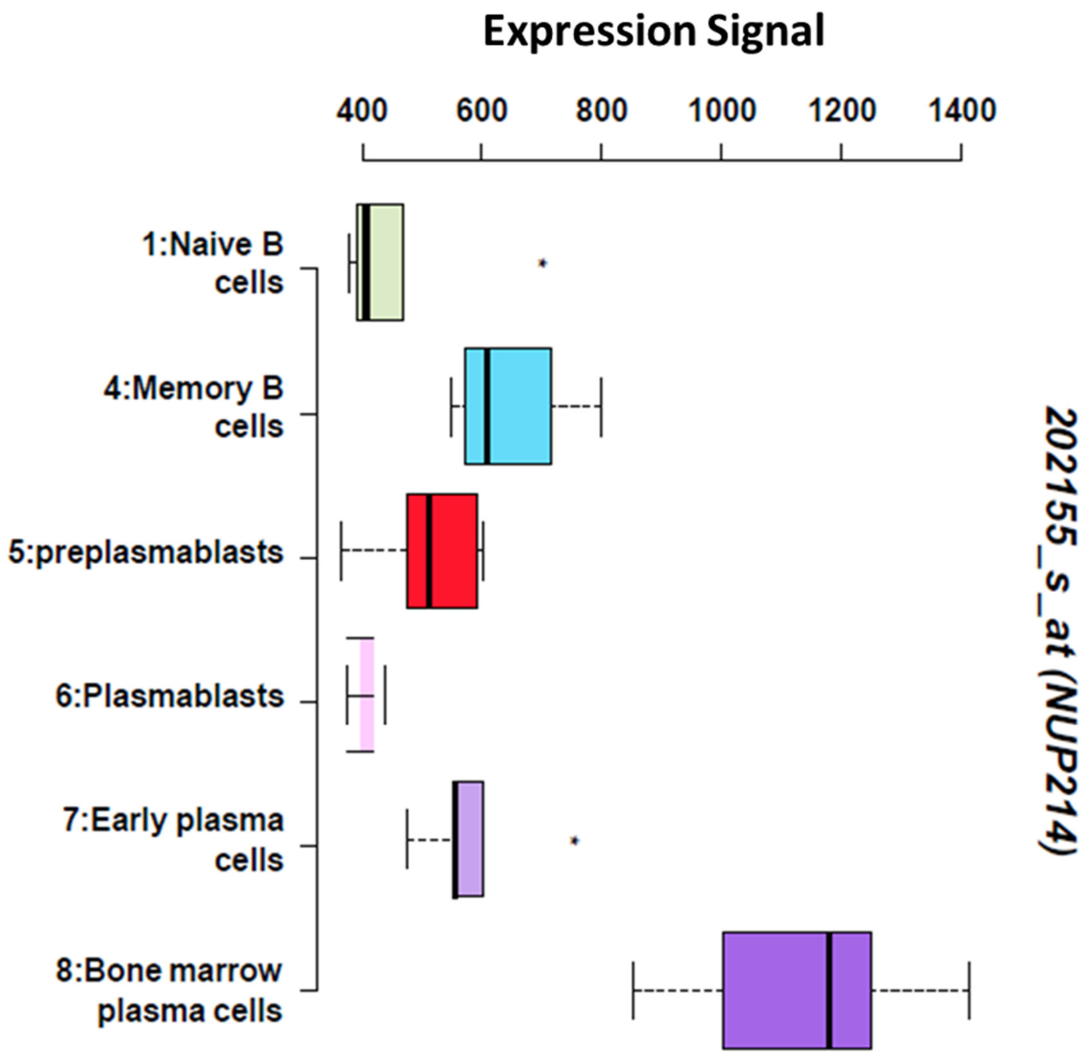

Expression Signal
400
600
800
1000
1200
1400
1:Naive B cells
4:Memory B cells
5:preplasmablasts
6:Plasmablasts
7:Early plasma cells
8:Bone marrow plasma cells
202155_s_at (NUP214)

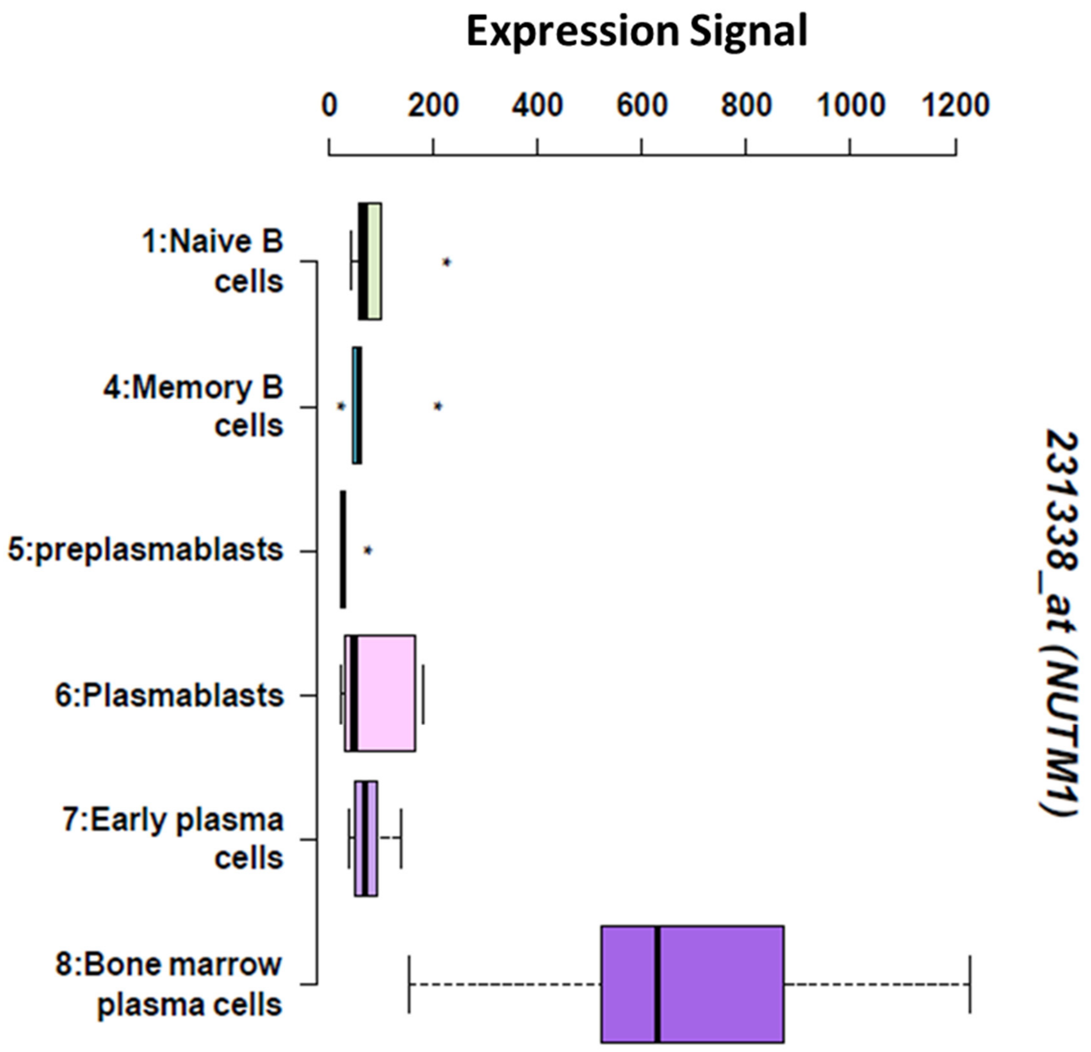
Expression Signal
0
200
400
600
800
1000
1200
1:Naive B cells
4:Memory B cells
5:preplasmablasts
6:Plasmablasts
7:Early plasma cells
8:Bone marrow plasma cells
231338_at (NUTM1)

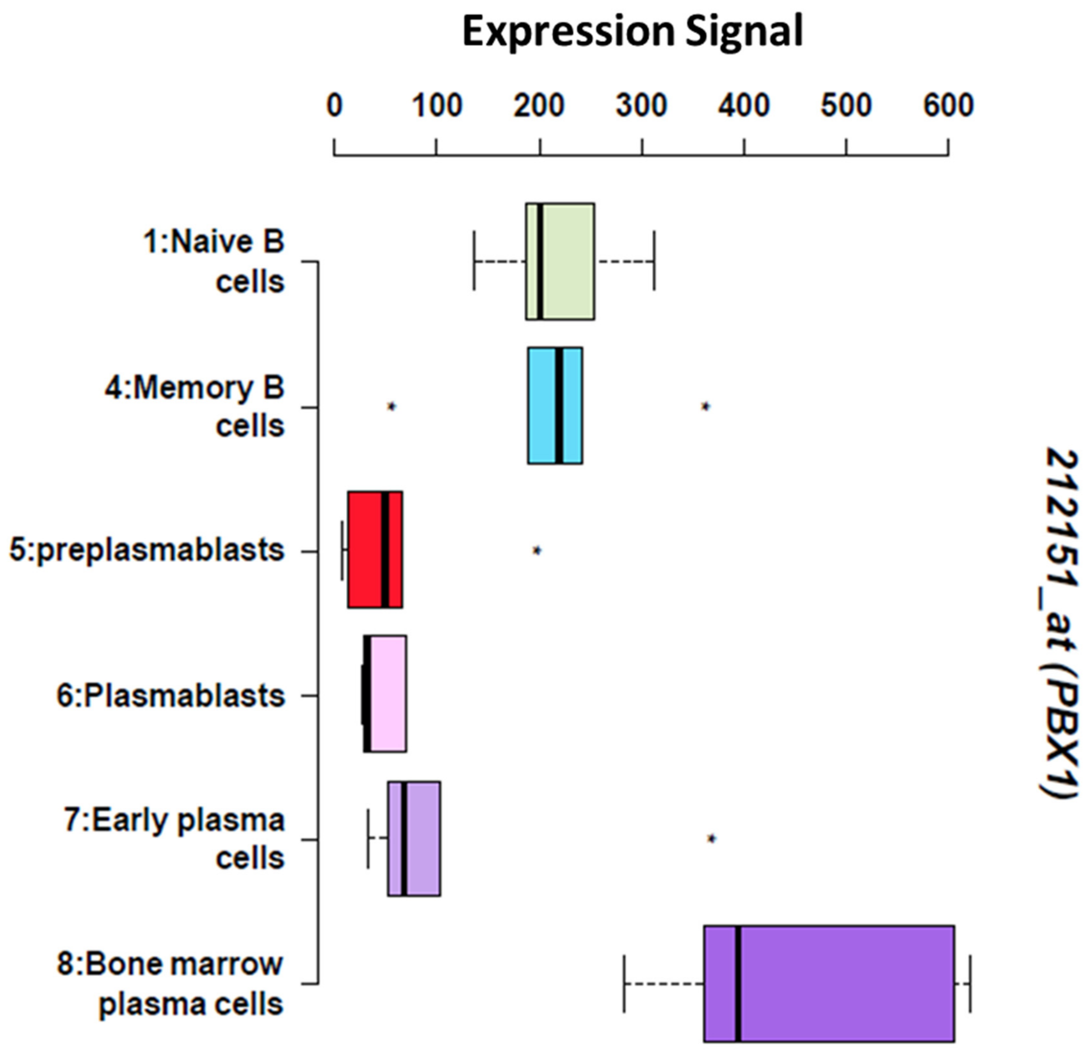

Expression Signal
0
100
200
300
400
500
600
1:Naive B cells
4:Memory B cells
5:preplasmablasts
6:Plasmablasts
7:Early plasma cells
8:Bone marrow plasma cells
212151_at (PBX1)

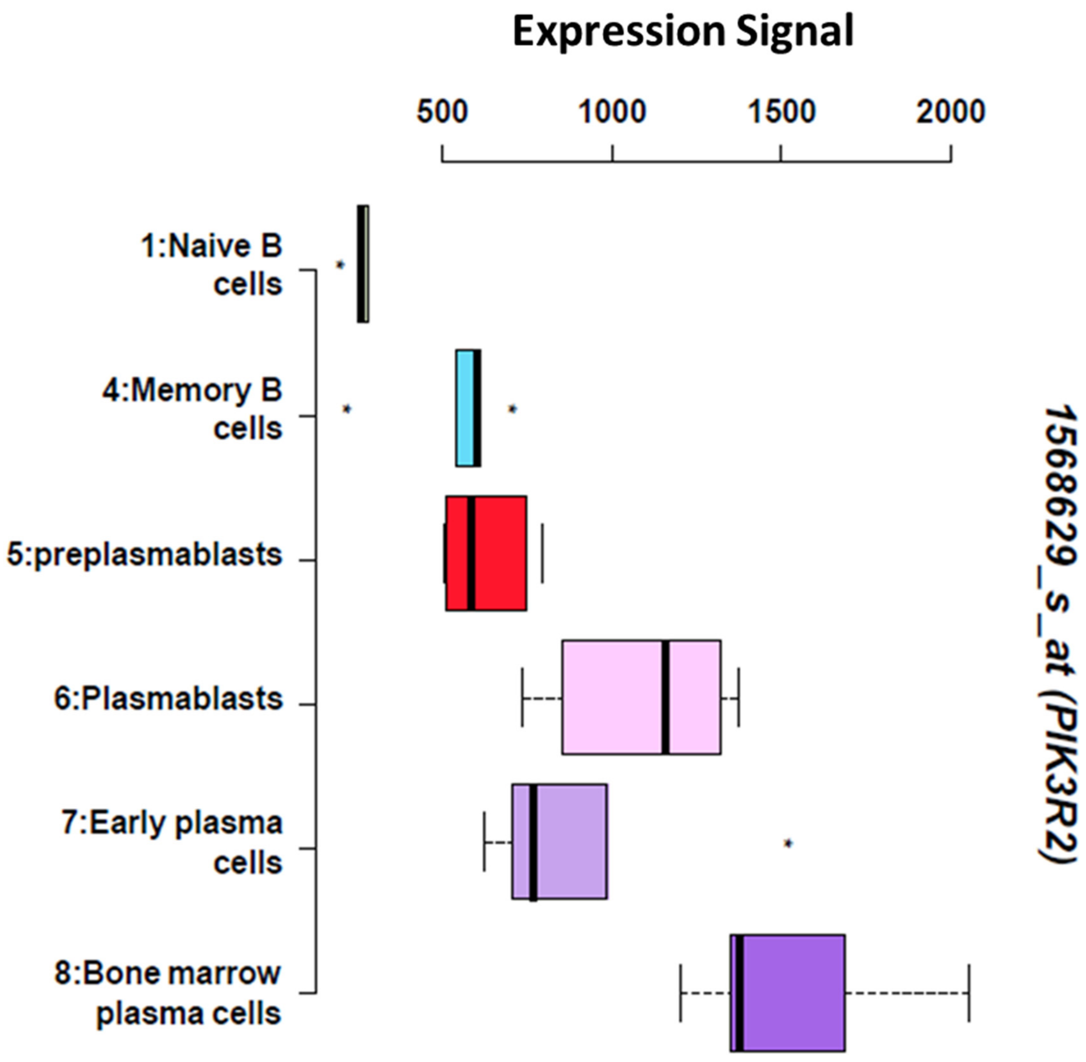

Expression Signal
500
1000
1500
2000
1:Naive B cells
4:Memory B cells
5:preplasmablasts
6:Plasmablasts
7:Early plasma cells
8:Bone marrow plasma cells
1568629_s_at (PIK3R2)

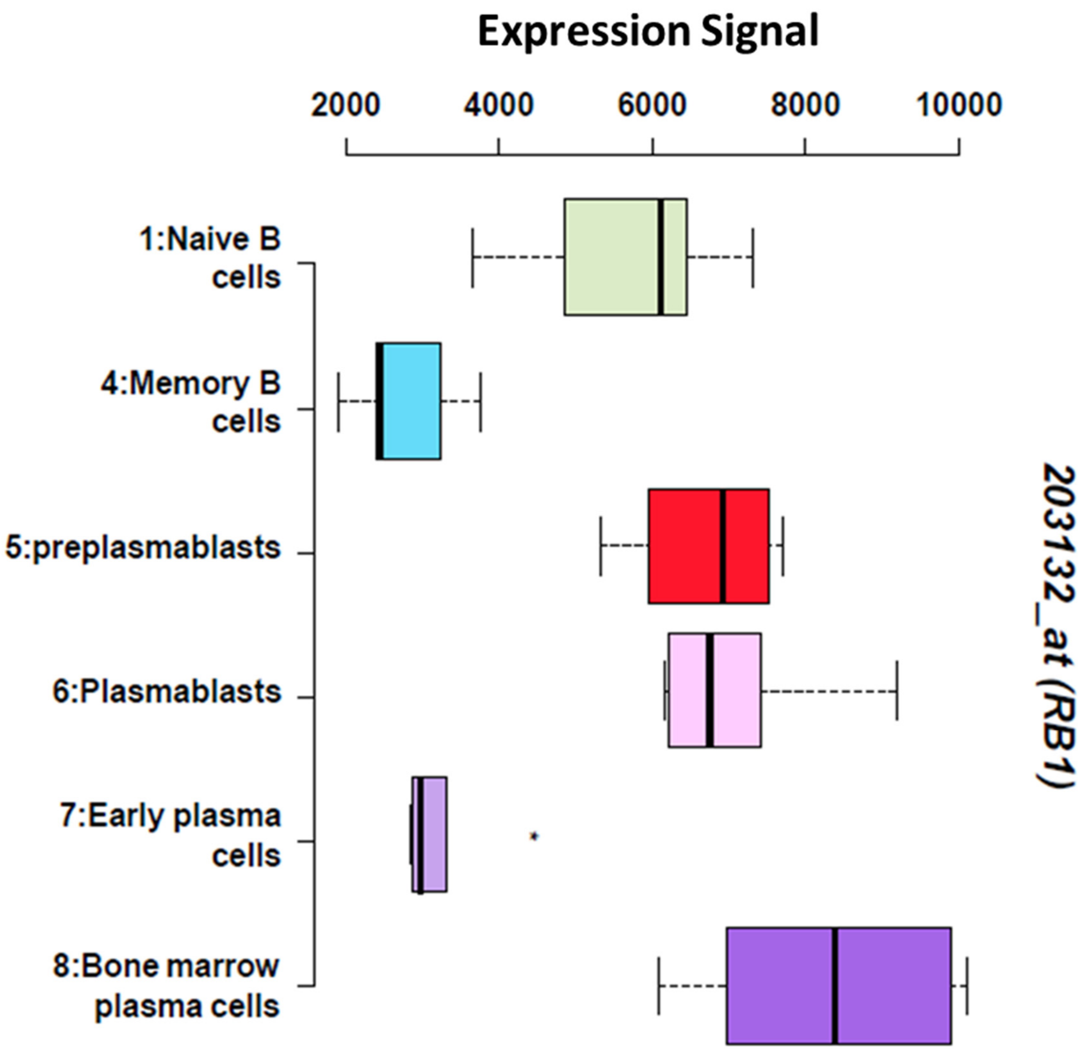

Expression Signal
2000
4000
6000
8000
10000
1:Naive B cells
4:Memory B cells
5:preplasmablasts
6:Plasmablasts
7:Early plasma cells
8:Bone marrow plasma cells
203132_at (RB1)

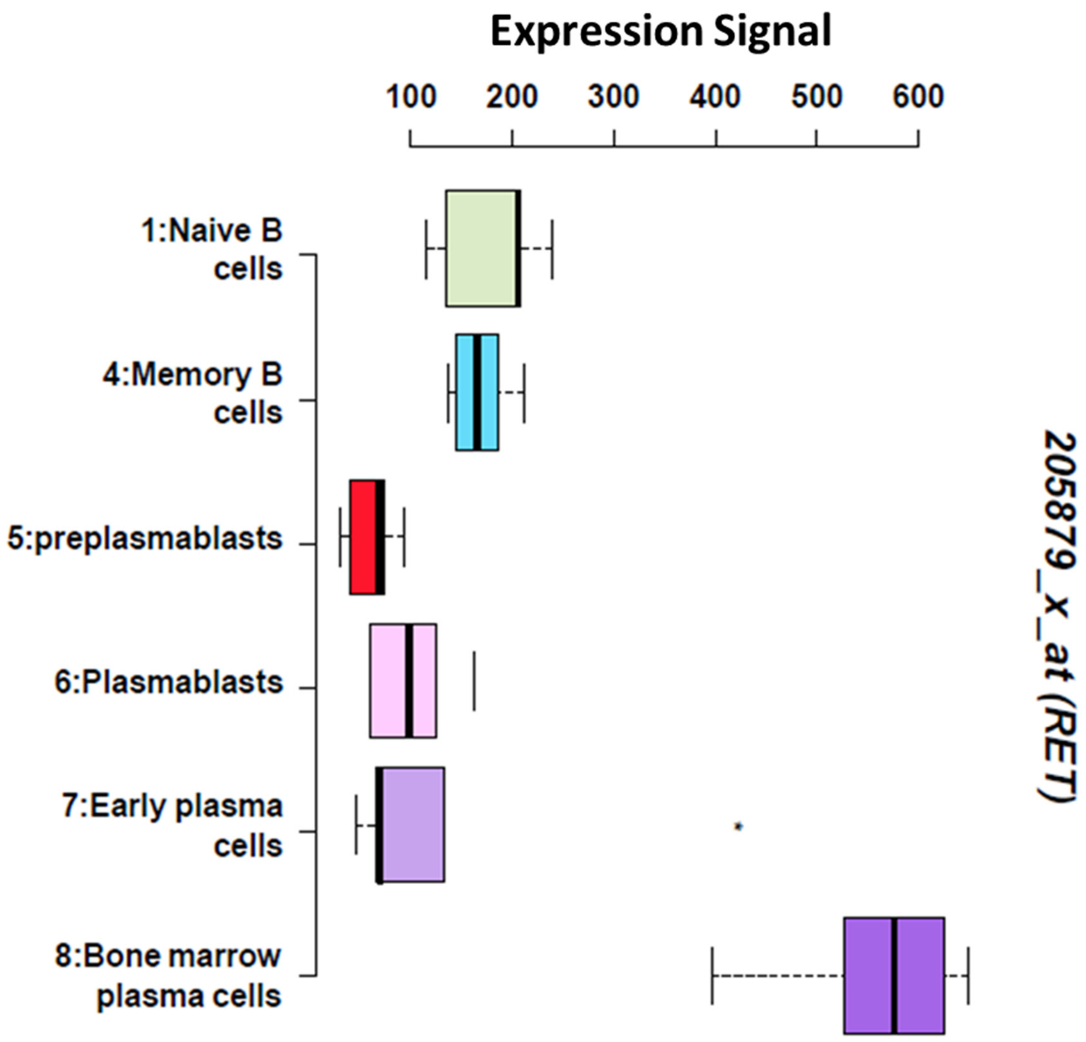
Expression Signal
100
200
300
400
500
600
1:Naive B cells
4:Memory B cells
5:preplasmablasts
6:Plasmablasts
7:Early plasma cells
8:Bone marrow plasma cells
205879_x_at (RET)

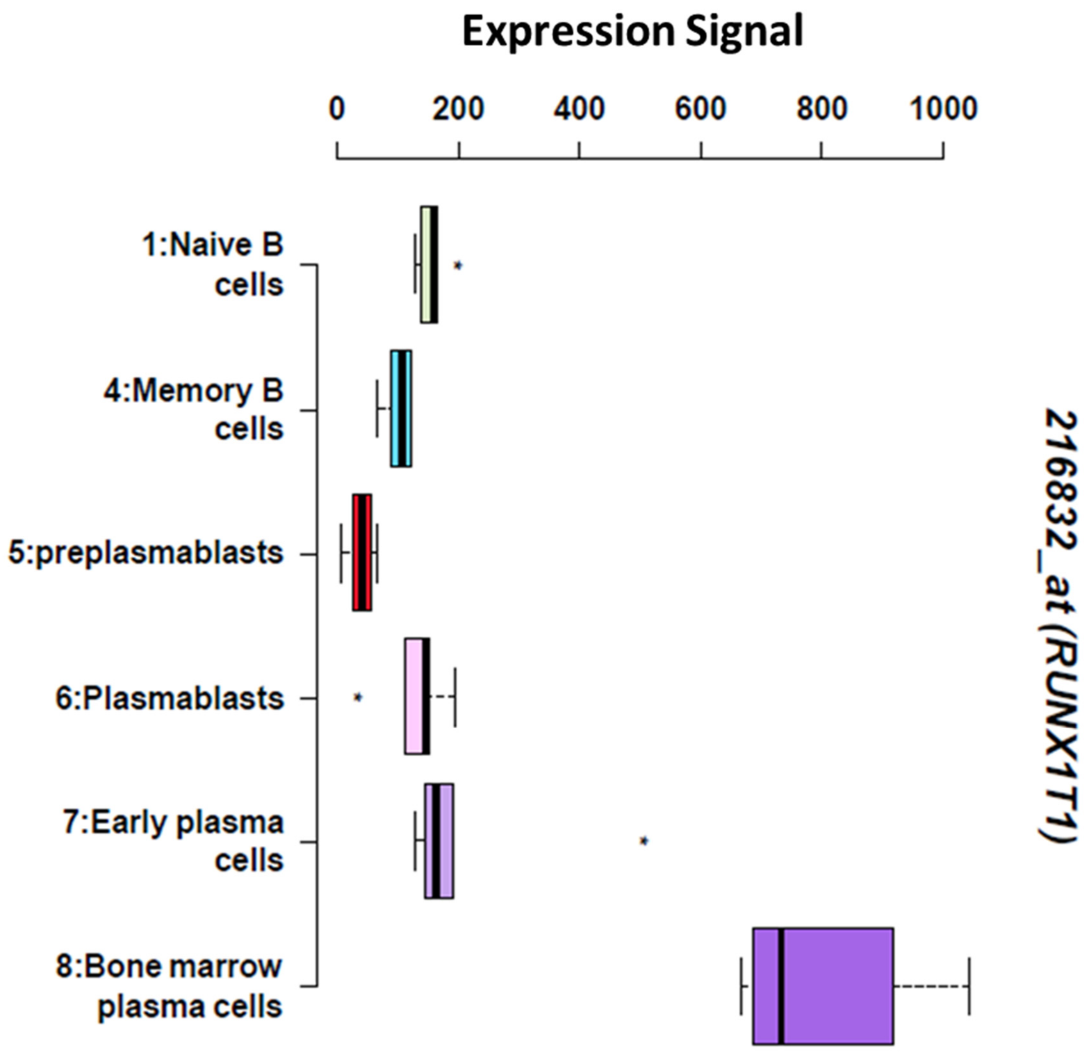
Expression Signal
0
200
400
600
800
1000
1:Naive B cells
4:Memory B cells
5:preplasmablasts
6:Plasmablasts
7:Early plasma cells
8:Bone marrow plasma cells
216832_at (RUNX1T1)

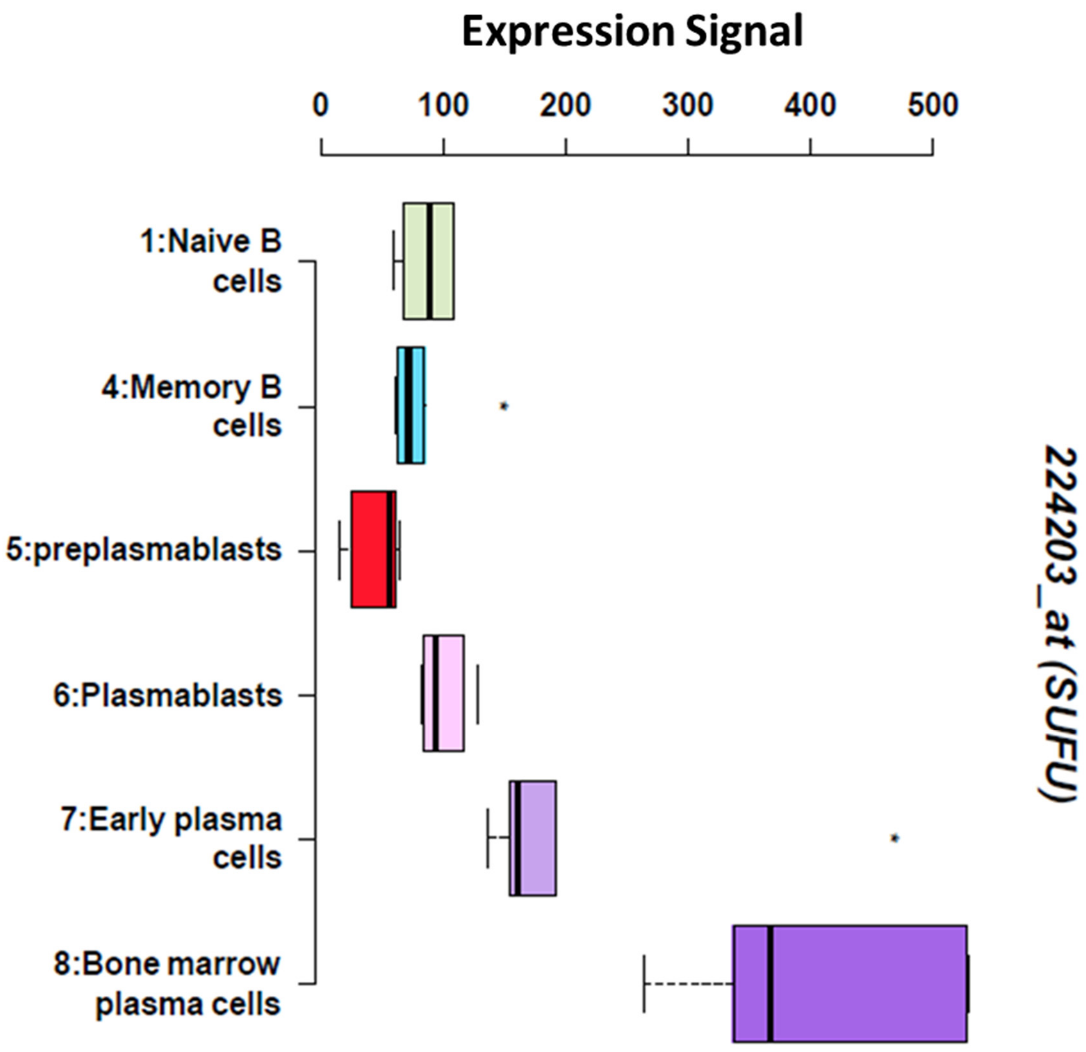
Expression Signal
0
100
200
300
400
500
1:Naive B cells
4:Memory B cells
5:preplasmablasts
6:Plasmablasts
7:Early plasma cells
8:Bone marrow plasma cells
224203_at (SUFU)

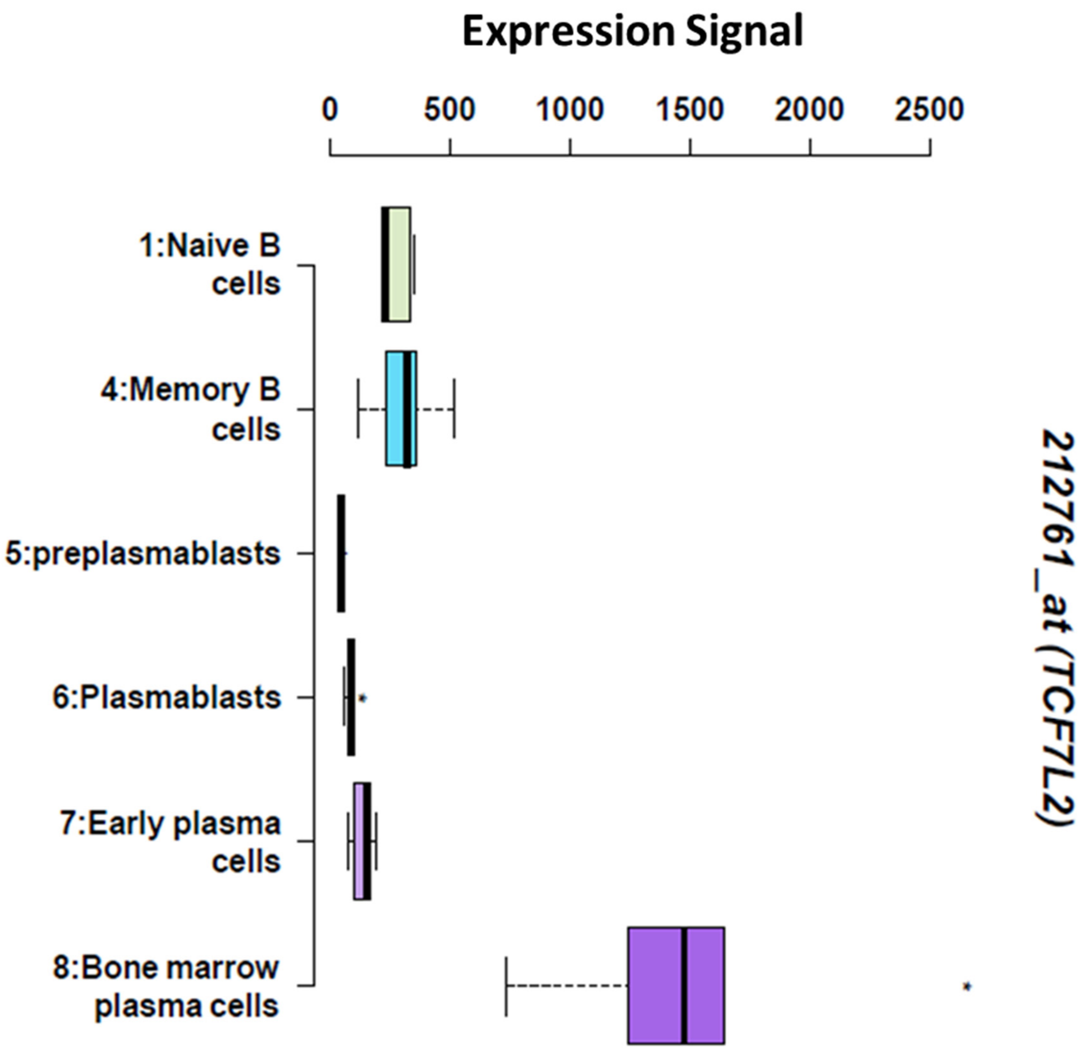
Expression Signal
0
500
1000
1500
2000
2500
1:Naive B cells
4:Memory B cells
5:preplasmablasts
6:Plasmablasts
7:Early plasma cells
8:Bone marrow plasma cells
212761_at (TCF7L2)

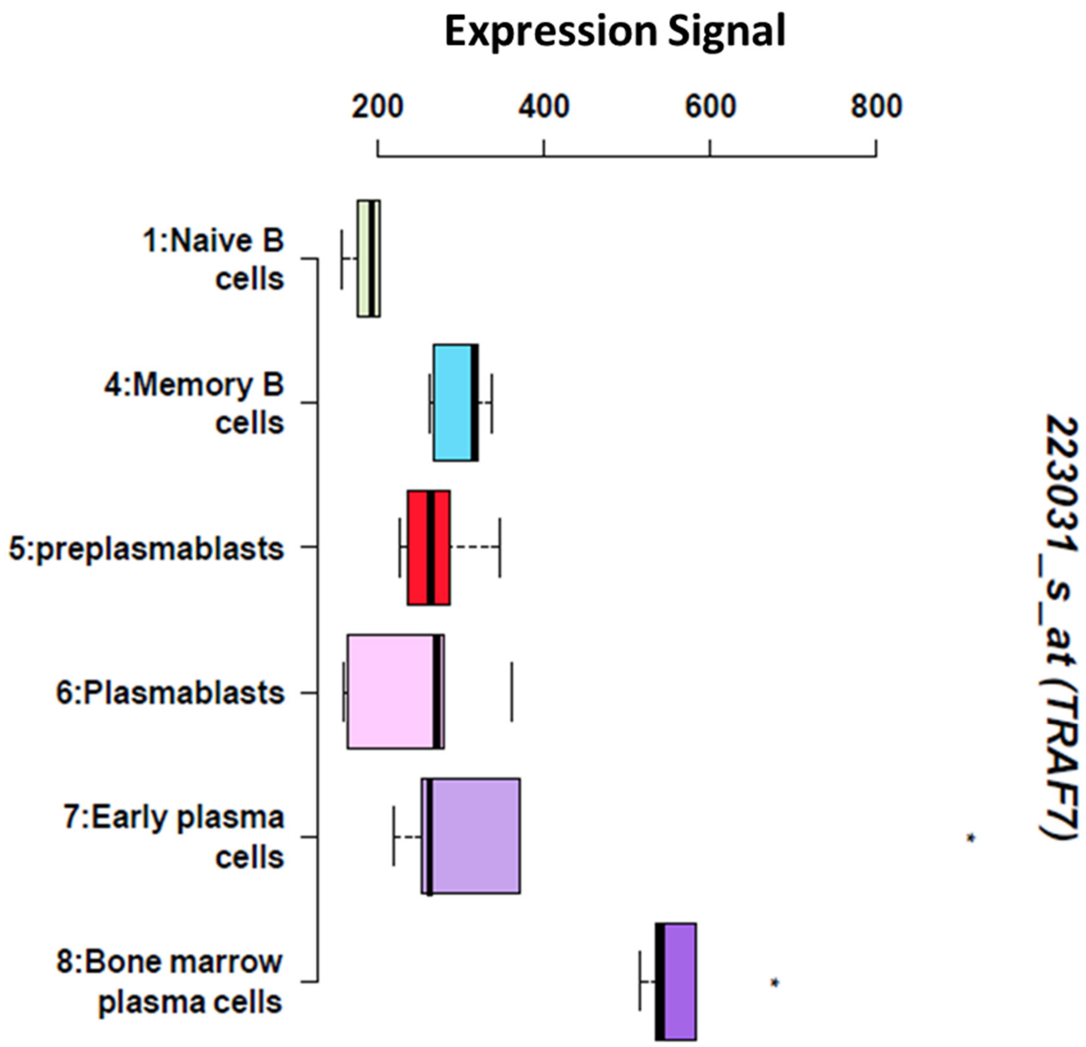
Expression Signal
200
400
600
800
1:Naive B cells
4:Memory B cells
5:preplasmablasts
6:Plasmablasts
7:Early plasma cells
8:Bone marrow plasma cells
223031_s_at (TRAF7)

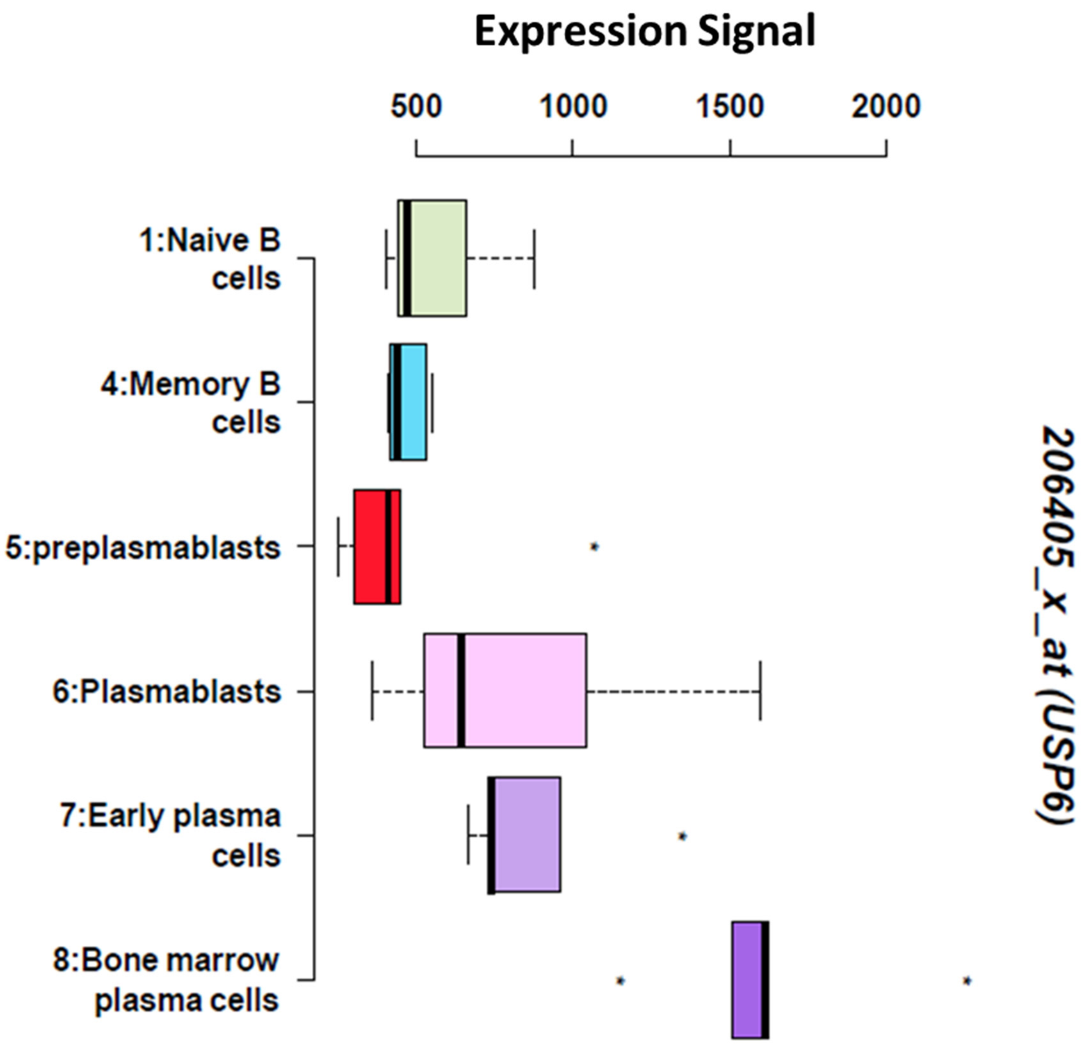

Figure S1. 33 genes frequently mutated in MM are overexpressed in bone marrow plasma cells(BMPCs). Expression of 33 genes frequently mutated in MM that are significantly overexpressed in BMPCs, inthe different subpopulations of the human plasma cell differentiation. Affymetrix microarrays expression box-plots were defined using GenomicScape web tool (http://www.genomicscape.com accessed on 23 July 2021). * indicates a significant difference using Student's *t*-test ($p \leq 0.05$).

Table S1. 83 TRCs resolution genes identified in the literature.

PROBESET	NAME
219841_at	AICDA
226320_at	ALYREF
1552937_s_a	ATRIP
205733_at	BLM
204531_s_at	BRCA1
208368_s_at	BRCA2
235609_at	BRIP1
218590_at	C10orf2
214426_x_at	CHAF1A
205394_at	CHEK1
202560_s_at	CHTOP
201241_at	DDX1
208159_x_at	DDX11
202577_s_at	DDX19A
224654_at	DDX21
201440_at	DDX23
220890_s_at	DDX47
225886_at	DDX5
223139_s_at	DHX36
202420_s_at	DHX9
214194_at	DIS3
213647_at	DNA2
218482_at	ENY2
235399_at	ERCC2
202176_at	ERCC3
235215_at	ERCC4
202414_at	ERCC5
204603_at	EXO1
207541_s_at	EXOSC10
209527_at	EXOSC2
227916_x_at	EXOSC3
218481_at	EXOSC5
212627_s_at	EXOSC7
203678_at	FAN1
203805_s_at	FANCA
242560_at	FANCD2
213007_at	FANCI
234733_s_at	FANCM
217902_s_at	HERC2
209330_s_at	HNRNPD

Table S2. 41 TRCs resolution genes significantly overexpressed in pre-plasmablasts comparedto memory B cells, plasmablasts and plasma cells.

PROBESET	NAME
219841_at	AICDA
226320_at	ALYREF
205733_at	BLM
204531_s_at	BRCA1
235609_at	BRIP1
218590_at	C10orf2
214426_x_at	CHAF1A
201241_at	DDX1
201440_at	DDX23
213647_at	DNA2
218482_at	ENY2
204603_at	EXO1
207541_s_at	EXOSC10
209527_at	EXOSC2
227916_x_at	EXOSC3
212627_s_at	EXOSC7
203805_s_at	FANCA
242560_at	FANCD2
213007_at	FANCI
209330_s_at	HNRNPD
200594_x_at	HNRNPU
205395_s_at	MRE11A
219940_s_at	PCID2
201202_at	PCNA
228252_at	PIF1
204354_at	POT1
217786_at	PRMT5
208393_s_at	RAD50
203344_s_at	RBBP8
212917_x_at	RECQL
218497_s_at	RNASEH1
203022_at	RNASEH2A
201528_at	RPA1
227447_at	SKIV2L2
201742_x_at	SRSF1
204064_at	THOC1
209418_s_at	THOC5
218848_at	THOC6
203046_s_at	TIMELESS
205672_at	XPA
223002_s_at	XRN2
